# Early alterations in a mouse model of Rett syndrome: the GABA developmental shift is abolished at birth

**DOI:** 10.1038/s41598-019-45635-9

**Published:** 2019-06-25

**Authors:** N. Lozovaya, R. Nardou, R. Tyzio, M. Chiesa, A. Pons-Bennaceur, S. Eftekhari, T.-T. Bui, M. Billon-Grand, J. Rasero, P. Bonifazi, D. Guimond, J.-L. Gaiarsa, D. C. Ferrari, Y. Ben-Ari

**Affiliations:** 1 Neurochlore, Ben-Ari Institute of Neuroarcheology (IBEN), Bâtiment Beret-Delaage, Parc scientifique et technologique de Luminy, 13288, Marseille, cedex 09 France; 2 Mediterranean Institute of Neurobiology (INMED), Department of Neurobiology, Aix-Marseille University, INSERM U1249, 13273, Marseille, France; 3 Biocruces Health Research Institute, 48903 Barakaldo, Spain; 4 IKERBASQUE: The Basque Foundation for Science, 48013 Bilbao, Spain

**Keywords:** Neurological disorders, Neuroscience

## Abstract

Genetic mutations of the Methyl-CpG-binding protein-2 (MECP2) gene underlie Rett syndrome (RTT). Developmental processes are often considered to be irrelevant in RTT pathogenesis but neuronal activity at birth has not been recorded. We report that the GABA developmental shift at birth is abolished in CA3 pyramidal neurons of Mecp2^−/y^ mice and the glutamatergic/GABAergic postsynaptic currents (PSCs) ratio is increased. Two weeks later, GABA exerts strong excitatory actions, the glutamatergic/GABAergic PSCs ratio is enhanced, hyper-synchronized activity is present and metabotropic long-term depression (LTD) is impacted. One day before delivery, maternal administration of the NKCC1 chloride importer antagonist bumetanide restored these parameters but not respiratory or weight deficits, nor the onset of mortality. Results suggest that birth is a critical period in RTT with important alterations that can be attenuated by bumetanide raising the possibility of early treatment of the disorder.

## Introduction

Rett syndrome (RTT) [**O**nline **M**endelian **I**nheritance in **M**an (OMIM) #312750] is a monogenic, severe disorder due to a genetic mutation of the Methyl-CpG-binding protein-2 (MECP2) gene that affects brain development with an incidence of 1 girl in 10,000–15,000 live births^1^. In addition, even though first considered lethal in the male population, few studies now report this mutation in male patients^2–4^. Symptoms include social withdrawal, frequent seizures, loss of purposeful hand use and expressive language, gait ataxia, mental retardation and respiratory distress^1,5^. Yet, whether a normal early development is followed by a clinical picture is controversial. Indeed, experimental studies using RTT mouse models (Mecp2-null or mice with an adult-onset of RTT) suggest that development might not be involved in the history of the disease^6–8^. However, it is now clear that RTT girls have many early pathological signs including abnormal general movements, tongue protrusion, postural stiffness, asymmetric eye opening and closing, hand stereotypies and bursts of abnormal face distortions^9–11^, some that may be present at birth^12–14^. In keeping with this, experimental studies show early gait and sensory and cortical organisation abnormalities in animal models^15^. Yet, in spite of this, we do not know whether these alterations are associated with neuronal electrical abnormalities that may already be present at birth.

Labor and birth are highly complex events in mammals associated with numerous biological changes. There is a release of hormones required to facilitate lungs maturation and the transition from a liquid medium to an aerial one^16–18^. Major alterations of the cardiovascular^19^, immune^20,21^ and microbiotic^22^ systems also take place. Hence, labor and birth are highly critical periods^23^ that when perturbed are associated with many neurodevelopmental disorders^24,25^. In addition, a fundamental issue is whether developmental processes are altered by perinatal insults notably during labor and birth^26–29^. Here, we have investigated whether neuronal activity is already impacted at birth in a mouse model of RTT. We focused on the GABA developmental shift as birth is associated with an abrupt neuroprotective oxytocin-mediated hyperpolarizing GABA action in pyramidal neurons^30^, shift that is abolished in the Fragile X (FRX) and intrauterine Valproic Acid (VPA) rodent models of Autism Spectrum Disorders (ASD)^31^. These alterations that persist in juvenile offspring are alleviated by maternal administration of the NKCC1 specific antagonist bumetanide. In addition, bumetanide treatment also attenuates behavioral sequels, suggesting that an alteration of the GABA response at birth is a marker of early abnormalities^31,32^. We now report similar alterations of intracellular chloride concentrations ([Cl^−^]_i_) in a mouse model of RTT that persist in juvenile offspring and are, at least in part, attenuated by bumetanide maternal pretreatment. Results suggest that the Mecp2 mutation impacts neuronal activity at birth with an abolition of the oxytocin-mediated GABA hyperpolarizing shift, stressing its importance in the pathogenesis of RTT.

## Results

### The GABA developmental shift is abolished in RTT

**Figure 1:**
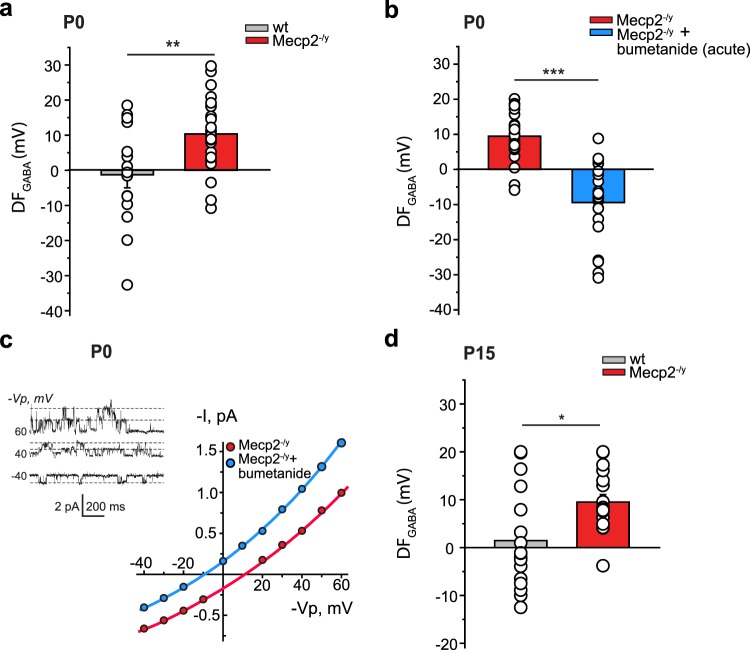
Depolarizing GABA signaling in hippocampal CA3 pyramidal neurons of Mecp2^−/y^ newborn and P15 mice. (**a**) Average values of DF_GABA_ at P0 in wt and Mecp2^−/y^ neurons. (**b**) Average values of DF_GABA_ at P0 in control condition and in the presence of bumetanide (10 µM) in Mecp2^−/y^ neurons. (**c**) Representative I/V curves of hippocampal CA3 pyramidal cells at P0 using cell-attached recordings of single GABA_A_R channels for the estimation of the DF_GABA_ in a representative Mecp2^−/y^ and Mecp2^−/y^ + bumetanide acute neuron. Inset shows single-channel GABA_A_R-mediated currents recorded at imposed voltages (−Vp, mV) in a Mecp2^−/y^ P0 pyramidal cell. Dashed lines show the average values of channel openings. Note the two levels of openings at 40 and 60 mV. (**d**) Average values of DF_GABA_ in wt and Mecp2^−/y^ neurons at P15. Data are presented as mean ± SEM. *p < 0.05; **p < 0.01; ***p < 0.001. (**a**,**b**,**d**) Significance was determined by two-tailed t-test. (see Supplementary Table 1 for detailed statistics).

We first focused on the developmental sequence of GABA_A_ receptors (GABA_A_Rs) currents polarity that is impaired in rodent models of early-life seizures, ASD, FRX and adult RTT^31,33–35^. In naive rodents, the driving force of GABAARs-mediated currents (DF_GABA_) is depolarizing in fetal hippocampal CA3 pyramidal neurons shortly before birth, and reduced to slightly depolarizing values at postnatal days 15 to 30 (P15 to P30), with an abrupt oxytocin-mediated hyperpolarization during the delivery period^30,31^. In keeping with this, the DF_GABA_ for wild-type (wt) mice was slightly hyperpolarizing at P0 in CA3 pyramidal neurons (Fig. 1a). In contrast, in Mecp2^−/y^ mice, the DF_GABA_ was elevated at birth, being significantly more depolarizing than for age-matched wt neurons (Fig. 1a and Supplementary Table 1). These differences are due to higher [Cl^−^]_i_ in neurons in Mecp2^−/y^ mice since bath application of the NKCC1 chloride importer antagonist bumetanide abolished the depolarizing DF_GABA_ leading to strongly hyperpolarizing values at P0 (Fig. 1b,c and Supplementary Table 1). Interestingly, this difference was not restricted to birth as the DF_GABA_ remained elevated two weeks later in Mecp2^−/y^ mice (P15, Fig. 1d and Supplementary Table 1). In addition, the quantification of overall fluorescence intensity of KCC2 labeling determined by immunohistochemistry revealed a significant reduction of KCC2 expression in the CA3 stratum pyramidale in Mecp2^−/y^ slices compared to the wt ones at P15 (Supplementary Fig. 1a,b and Table 1). Therefore, a persistent increase of [Cl^−^]_i_ is observed in Mecp2^−/y^ neurons starting from birth and maintained at P15.

### GABA excitatory actions in P15 Mecp2^−/y^ mice are rescued by maternal pretreatment with bumetanide

**Figure 2:**
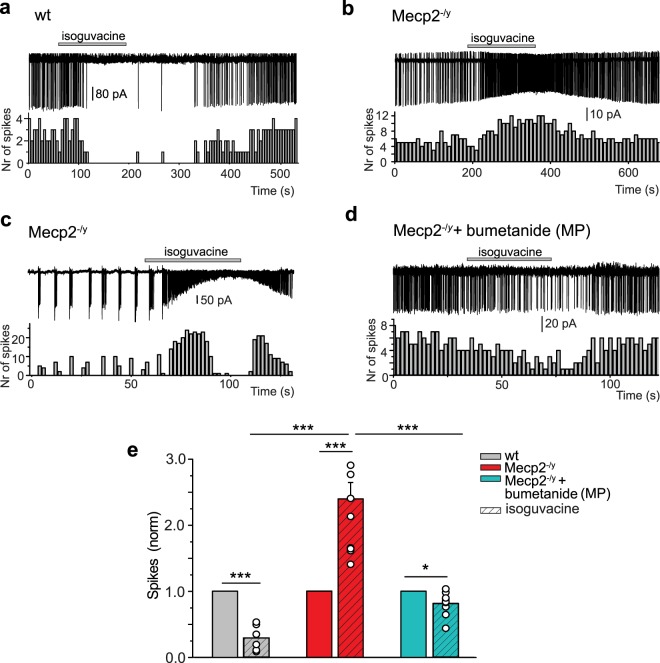
Excitatory action of GABA in hippocampal CA3 pyramidal neurons in P15 Mecp2^−/y^ mice is rescued by maternal pretreatment with bumetanide. Inhibitory action in wt (**a**) and excitatory action in Mecp2^−/y^ (**b**,**c**) of the GABA_A_R agonist isoguvacine (10 μM) on spontaneous spikes recorded in cell-attached configuration. (**c**) Example of a “bursty” spiking CA3 hippocampal pyramidal cell activity in Mecp2^−/y^ mice. (**d**) Restored inhibitory action of isoguvacine in Mecp2^−/y^ mice with bumetanide maternal pretreatment (MP). (**a**–**d**) Time-course of spike frequency changes is shown under each trace. (**e**) Mean values of isoguvacine effects normalized to baseline spike frequency in wt, Mecp2^−/y^ mice, and Mecp2^−/y^ mice with MP bumetanide. Data are presented as mean ± SEM. *p < 0.05; ***p < 0.001. (**e**) Datasets analyzed by paired sample two-tailed t-test and One-way ANOVA with Fisher’s least significant difference as a post hoc test. (see Supplementary Table 2 for detailed statistics).

**Figure 3:**
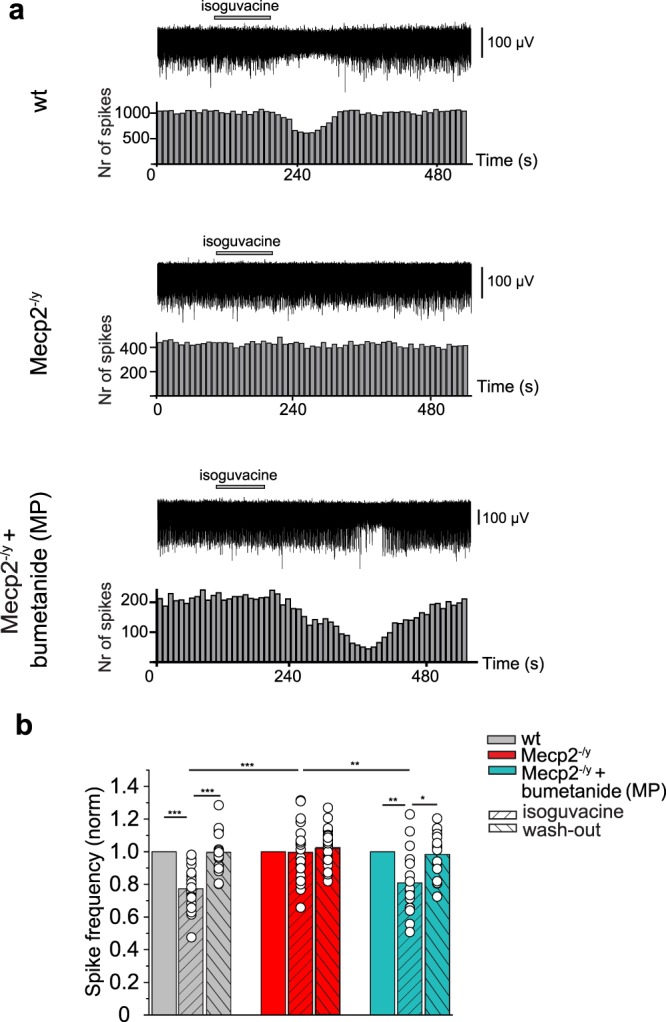
Maternal pretreatment with bumetanide before delivery restores inhibitory GABA actions in Mecp2^−/y^ mice at P16. (**a**) Representative traces of spontaneous extracellular field potentials recorded in the CA3 zone of hippocampal slices, and effects of isoguvacine (10 μM) at P16 in wt, Mecp2^−/y^ mice, and bumetanide pretreated (MP) Mecp2^−/y^ mice. Corresponding time courses of spike frequency changes are shown under traces. (**b**) Mean values of isoguvacine effects normalized to baseline spike frequency in wt, Mecp2^−/y^, and Mecp2^−/y^ MP mice. Data are presented as mean ± SEM. *p < 0.05; **p < 0.01; ***p < 0.001. (**b**) Data set analyzed by repeated measures ANOVA with Tukey’s post-hoc test and one-way ANOVA with Tukey’s post-hoc test (see Supplementary Table 3 for detailed statistics).

In ASD rodent models, like in a wide range of disorders, high [Cl^−^]_i_ is associated with excitatory GABA actions since application of GABA agonists generates action potentials or increases the frequency of action potentials^31,36^. We therefore tested the effects of the GABA_A_Rs agonist isoguvacine (10 μM) application on CA3 pyramidal neurons at P15 in cell-attached patch clamp recording. In wt neurons, isoguvacine transiently reduced ongoing spike frequency (Fig. 2a,e and Supplementary Table 2). In contrast, isoguvacine significantly increased the frequency of action potentials in age-matched Mecp2^−/y^ neurons (Fig. 2b,c,e and Supplementary Table 2). In many Mecp2^−/y^ neurons, ongoing activity was characterized by the presence of bursting activity (Fig. 2c). Isoguvacine abolished these bursts that were replaced by high frequency ongoing activity. We then tested the hypothesis that restoring low [Cl^−^]_i_ and inhibitory GABA actions specifically during delivery rescues immature electrophysiological features in juvenile offspring. We treated pregnant mice with bumetanide in the drinking water for 1 day at E18 and tested in offspring the effects of isoguvacine at P15. Thus, a single treatment with bumetanide around birth transformed the GABA action recorded in the two-weeks old offspring from excitation to inhibition, as revealed by a reduction of the spike frequency (Fig. 2d,e and Supplementary Table 2). Similar experiments were made with extracellular field potential recordings from the CA3 zone of hippocampal slices (Fig. 3 and Supplementary Table 3). Isoguvacine inhibited spontaneous field potential activity in wt slices but did not affect the frequency of spontaneous field potential activity in Mecp2^−/y^ slices. These effects differ from the excitation produced by isoguvacine in cell-attached recordings in Mecp2^−/y^ slices (Fig. 2e); these differences are likely due to the heterogeneity of the population recorded with field recordings including pyramidal neurons but also various types of interneurons. Nevertheless, maternal pretreatment with bumetanide induced GABA inhibitory activity in the Mecp2^−/y^ P15 hippocampal network.

### Sequential alterations of glutamatergic and GABAergic spontaneous hippocampal network activities in Mecp2^−/y^ mice

**Figure 4:**
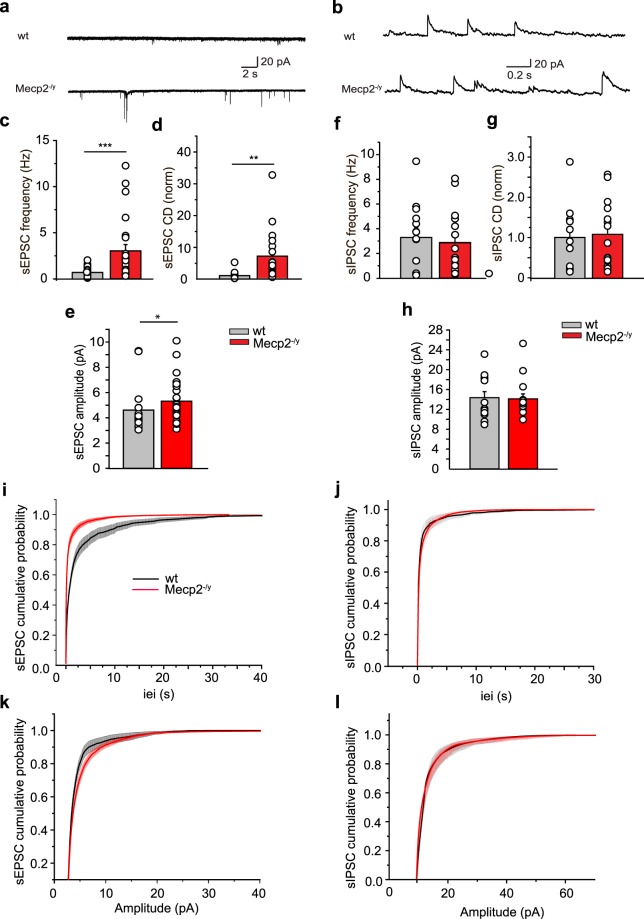
Spontaneous glutamatergic activity is already increased in Mecp2^−/y^ mice at birth. Representative traces of whole-cell voltage clamp recordings of (**a**) sEPSC at −75 mV and (**b**) sIPSC at + 10 mV from individual hippocampal CA3 pyramidal neurons in hippocampal slices from wt and Mecp2^−/y^ mice at P0. (**c**–**h**) Average values of sEPSC (**c**–**e**) and sIPSC (**f**–**h**) frequencies, amplitudes and charge densities (CD, ΣCharge per sec normalized to control) in wt and Mecp2^−/y^ mice at P0. (**i**–**l**) Cumulative distributions of sEPSC and sIPSC inter-event-interval (iei) and amplitude. Data are presented as mean ± SEM. *p < 0.05; **p < 0.01; ***p < 0.001. (**c**–**h**) Significance was determined by Mann-Whitney test (see Supplementary Tables 4 and 5 for detailed statistics).

**Figure 5:**
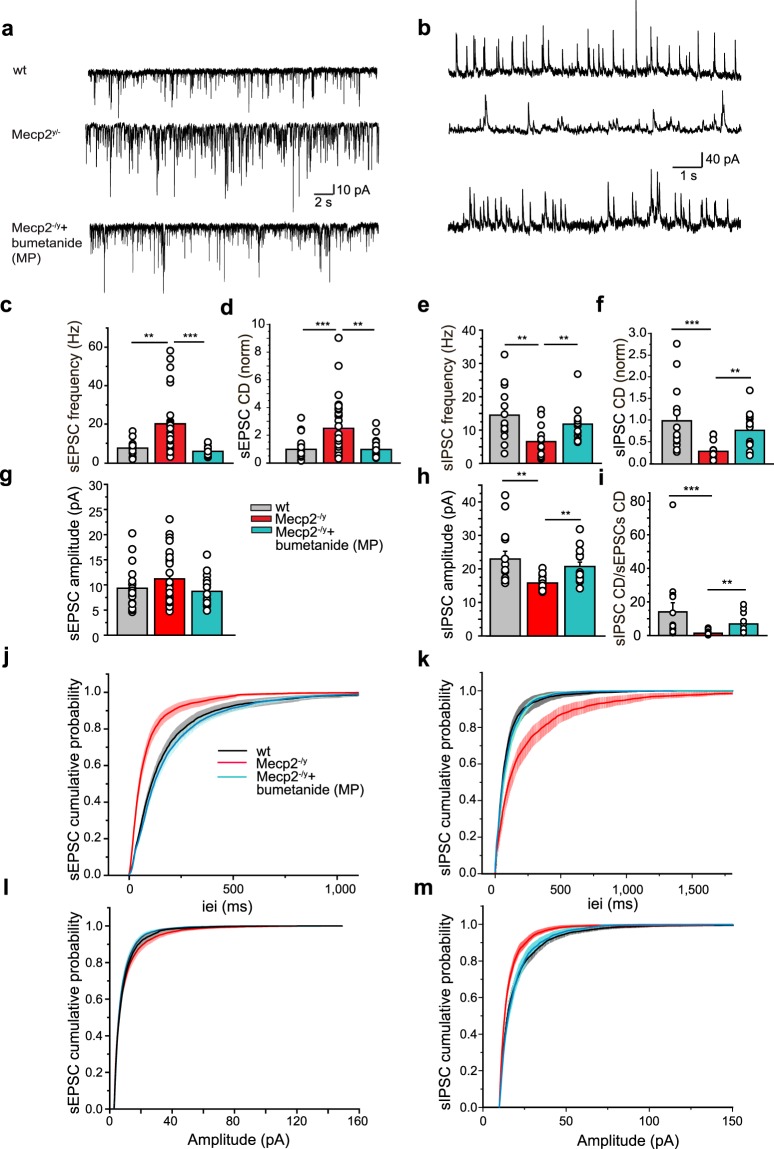
Excitation/inhibition ratio is increased in Mecp2^−/y^ mice at P15 and ameliorated by maternal pretreatment with bumetanide. Representative traces of (**a**) sEPSC and (**b**) sIPSC recorded respectively at −75 mV and + 10 mV from individual hippocampal CA3 pyramidal neurons in hippocampal slices from wt, Mecp2^−/y^ and bumetanide pretreated (MP) Mecp2^−/y^ mice at P15. Average values of sEPSC (**c,d,g**) and sIPSC (**e,f,h)** frequencies, amplitudes and charge densities (CD, ΣCharge per sec, normalized to control) in wt, Mecp2^−/y^ mice and Mecp2^−/y^ MP. (**i**) sIPSC/sEPSC CD ratio in wt, Mecp2^−/y^ mice with and without MP bumetanide. (**j–m**) Cumulative distributions of inter-event-interval (iei) and amplitude of sEPSC and sIPSC. Data are presented as mean ± SEM. **p < 0.01; ***p < 0.001. (**c–i**) Data were analyzed with Kruskal-Wallis test with Dunn’s multiple comparison post-hoc test (see Supplementary Tables 6-8 for detailed statistics).

The balance between excitation and inhibition is instrumental in the operation of brain networks and is disturbed in many disorders including ASD and RTT^31,34,35,37^. This alteration is mediated by either a reduction of inhibition and/or an increased glutamatergic drive. We therefore measured spontaneous GABAergic postsynaptic currents (sIPSC) and glutamatergic postsynaptic currents (sEPSC) at birth and P15 in order to estimate neuronal network excitability as well as the inhibitory/excitatory balance at both developmental stages. To isolate sEPSC from sIPSC, we held the membrane potential at the reversal potentials of inhibitory (−75 mV) and excitatory inputs (+10 mV), respectively. At birth, whole-cell recordings revealed that the frequency and amplitude of sEPSC were significantly increased in Mecp2^−/y^ mice (Fig. 4a,c,e,i,k and Supplementary Table 4). This was manifested by an increase of charge density (Fig. 4d and Supplementary Table 4). In contrast, neither the frequency, amplitude nor charge density of sIPSC appeared modified at P0 (Fig. 4b,f–h,j,l and Supplementary Table 5). A different situation prevailed at P15. Even though the amplitude of sEPSC was not found to be altered (Fig. 5g,l and Supplementary Table 6), the frequency was still significantly increased (Fig. 5a,c,j and Supplementary Table 6). Contrarily, the frequency and amplitude of sIPSC were reduced (Fig. 5b,e,h,k,m and Supplementary Table 7) in Mecp2^−/y^ mice. Estimation of the charge density of sEPSC and sIPSC revealed an increase of the former and a parallel decrease of the latter (Fig. 5d,f and Supplementary Tables 6, 7) leading to the dramatic net reduction of the sIPSC/sEPSC ratio at P15 (Fig. 5i and Supplementary Table 8). However, frequency and amplitude of miniature GABAergic and glutamatergic PSCs were similar between wt and Mecp2^−/y^ mice (Supplementary Figs 2a–c; 3a–c and Tables 9 and 10), suggesting that sEPSC and sIPSC alterations are network-mediated. Maternal bumetanide pretreatment restored the sIPSC/sEPSC ratio at P15 in Mecp2^−/y^ mice including the frequency, amplitude and charge density parameters (Fig. 5c–m and Supplementary Tables 6–8). Therefore, the excitation/inhibition balance is already altered at birth in Mecp2^−/y^ mice, it persists at P15 and is rescued by maternal bumetanide pretreatment.

### Alterations of spontaneous synapse-driven network oscillations in Mecp2^−/y^ mice

**Figure 6:**
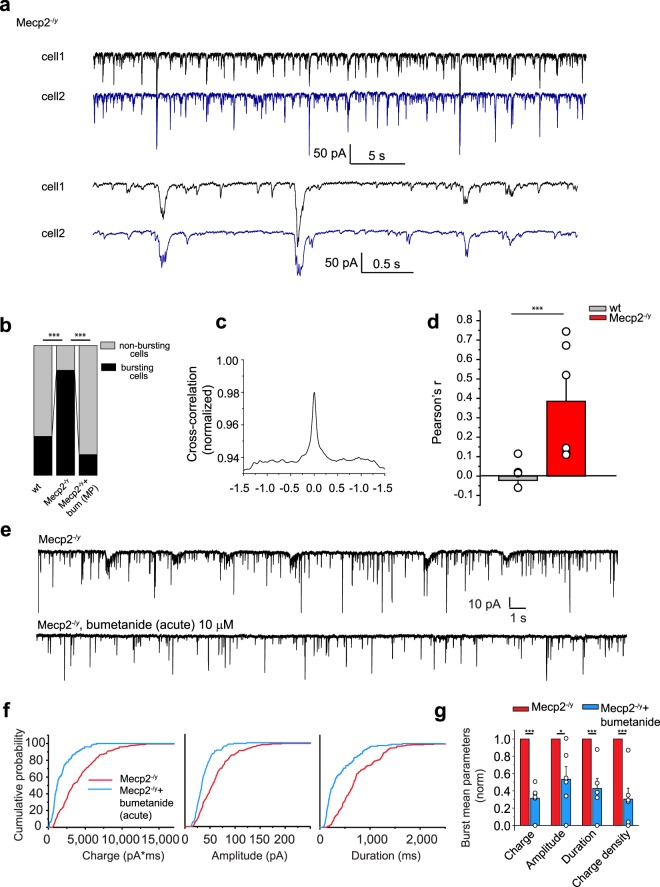
Hypersynchronization in hippocampal CA3 pyramidal neurons network in Mecp2^−/y^ at P15 is rescued by bumetanide. **(a**) Representative traces of dual whole-cell voltage clamp recordings of glutamatergic repetitive population bursts recorded at −75 mV from two neighboring hippocampal CA3 pyramidal neurons in hippocampal slices from P15 Mecp2^−/y^ mice. Lower traces are shown at extended timescale. (**b**) Relative contribution of cells displaying population bursts in wt, Mecp2^−/y^ and Mecp2^−/y^ with MP bumetanide represented in %. (**c**) Representative cross-correlation between sEPSC traces recorded in both cells shown in (**a**). (**d**) Averaged Pearson’s coefficient for dual sEPSC traces. (**e**) Representative traces of sEPSCs from “bursty” hippocampal CA3 pyramidal neurons recorded at −75 mV in Mecp2^−/y^ mice with and without acute bumetanide treatment. (**f**) Averaged cumulative probabilities of charge, amplitude and duration of bursts in brain slices of Mecp2^−/y^ and Mecp2^−/y^ with acute bumetanide treatment. (**g**) Burst mean parameters: charge, amplitude, duration and charge density in Mecp2^−/y^ and in Mecp2^−/y^ + bumetanide. Data are presented as mean ± SEM. *p < 0.05; ***p < 0.001. (**b**) Significance was determined with the two-tailed Fisher’s exact test with Bonferroni correction. (**d**) Pearson coefficient in wt and Mecp2^−/y^. (**g**) Datasets were analyzed with the paired sample two-tailed t-test. See Supplementary Table 11 for detailed statistics.

Spontaneous activity of CA3 pyramidal neurons in Mecp2^−/y^ mice is characterized by repetitive population bursts at P15 (Fig. 6a). Thus, 81% of Mecp2^−/y^ cells display glutamatergic population bursts compared with 30% in wt mice. After maternal pretreatment with bumetanide, only 17% of Mecp2^−/y^ cells displayed a “bursty” pattern (Fig. 6b and Supplementary Table 11).

To better understand the network alterations in Mecp2^−/y^ mice, we performed paired recordings from two nearby (≤200 µm) CA3 hippocampal pyramidal neurons. Glutamatergic repetitive population bursts were synchronized in pair-recorded Mecp2^−/y^ cells, as reflected by the Pearson’s correlation coefficient (0.39 ± 0.12 for Mecp2^−/y^ vs −0.022 ± 0.036 for wt mice) (Fig. 6a,c,d and Supplementary Table 11). These synchronized glutamatergic events were blocked by acute bumetanide application, restoring an ongoing activity that is reminiscent of that of wt age-matched neurons, thus suggesting that the excitatory actions of GABA acutely facilitate the generation of synchronized population events (Fig. 6e–g and Supplementary Table 11). Therefore, a high [Cl^−^]_i_-dependent aberrant synchronization is already present in pre-symptomatic Mecp2^−/y^ mice.

### Metabotropic LTD is altered in Mecp2^−/y^ mice and corrected by maternal pretreatment with bumetanide

**Figure 7:**
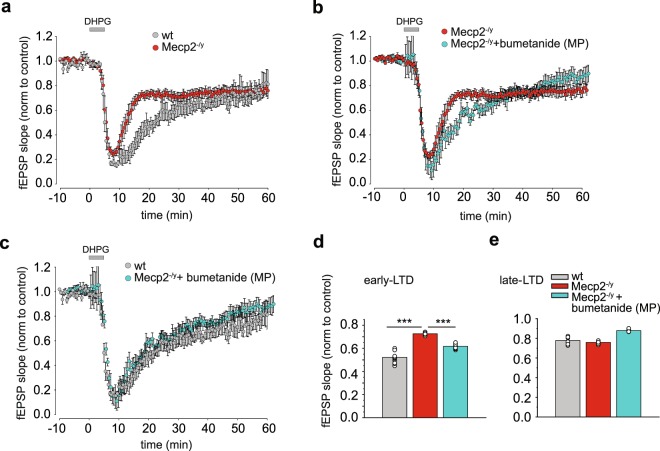
Maternal pretreatment with bumetanide rescues impaired mGluR-induced LTD in Mecp2^−/y^ mice at P15. **(a**) Early phase of DHPG-induced LTD (early –LTD) at the Schaffer Collaterals-CA1 synapses was attenuated in Mecp2^−/y^ mice 20–25 min following washout of drug, whereas slices from wt mice demonstrated a robust early-LTD. (**b**,**c**) Maternal pretreatment (MP) with bumetanide restored attenuated early-LTD in Mecp2^−/y^ mice. (**d**) Averaged fEPSP slope for early-LTD (time window from 20 to 25 min after DHPG washout) and late phase of DHPG-LTD (late-LTD) (time window from 50 to 55 min after DHPG washout) relative to averaged baseline values for wt, Mecp2^−/y^ and Mecp2^−/y^ MP with bumetanide mice. Data are presented as mean ± SEM. ***p < 0.001. Data set analyzed by One-way ANOVA with Fisher’s least significant difference post-hoc test (see Supplementary Table 12 for detailed statistics).

Common features of mice models of RTT are synaptic plasticity dysfunction, and deficits in learning and memory^38,39^. Indeed, impaired synaptic plasticity was observed in hippocampal slices from Mecp2-null mice and in cortical slices from Mecp2^308/y^ mice^40,41^, suggesting that these changes in synaptic plasticity consistently result from the loss of Mecp2. We therefore examined if the metabotropic glutamate receptors (mGluRs) agonist (*S*)-3,5-Dihydroxyphenylglycine (DHPG)-induced long-term depression (LTD) at the hippocampal Shaffer collaterals-CA1 synapse in P15 wt and Mecp2^−/y^ littermates^42^. In brain slices from wt mice, treatment with 50 µM DHPG induced an LTD response that was 77.83 ± 1.08% of the baseline fEPSP slope at 50-55 min following washout (we refer it later as late-LTD, Fig. 7a,e and Supplementary Table 12) and 52.18 ± 1.56% of the baseline fEPSP slope at 20–25 min following washout (referred to as early-LTD; Fig. 7a,d and Supplementary Table 12). The magnitude of the late-LTD was not significantly different between wt and Mecp2^−/y^ mice (Fig. 7a,e and Supplementary Table 12). However, early-LTD was significantly decreased in slices from Mecp2^−/y^ mice compared to wt, suggestive of a deficit in this form of synaptic plasticity (Fig. 7a,d and Supplementary Table 12). Interestingly, in keeping with our hypothesis, maternal pretreatment with bumetanide attenuated the early-LTD deficit in Mecp2^−/y^ mice (Fig. 7b,c,d and Supplementary Table 12). Therefore, LTD is already impacted in RTT at P15 and corrected by maternal pretreatment with bumetanide.

### Breathing deficiency in Mecp2^−/y^ mice is not rescued by bumetanide

Mecp2^−/y^ young-adult and adult mice have severe cardiorespiratory deficits with irregular breathing patterns and breath-holding, recapitulating human patients symptoms^43,44^. In addition, at P3-P5, breathing patterns and the number of medullary neurons expressing Tyrosine Hydroxylase (TH) are similar in wt and Mecp2^−/y^ mice, but breathing abnormalities are observed around 6 weeks of age and the number of TH-positive neurons in the medulla is significantly reduced in 2 months old mice^45^. Mecp2^−/y^ young-adult and adult mice also present low weight compared to controls as early as 4 weeks of age, and a short lifespan^6^. Therefore, we evaluated the breathing patterns of unrestrained mice with whole-body plethysmography along with bodyweight and onset of mortality to test whether bumetanide can rescue these deficiencies in Mecp2^−/y^ mice. We show that the cumulative probability distribution of the inter-breathing cycle-interval (ICI) of Mecp2^−/y^ mice differs from wt mice already at P24 (Supplementary Fig. 4a–c and Table 13). Measuring the distance between the ICI datasets in each pair of animals revealed high similarity (i.e. low distance) between animals within the wt group and within the Mecp2^−/y^ group independently of the maternal pretreatment, but not between the wt and the Mecp2^−/y^ mice (Supplementary Fig. 4c**)**. Unsupervised clustering analysis of all the mice studied at all ages into the 3 conditions (wt, Mecp2^−/y^ mice, and Mecp2^−/y^ + maternal pretreatment with bumetanide) identified with statistical significance (p < 0.01, see Methods) only two groups corresponding to the wt group and to Mecp2^−/y^ group with no distinction between treated and untreated animals (Supplementary Fig. 4d and Table 13**)**. We further show that Mecp2^−/y^ mice were underweight compared to their wt littermates starting at 3.5 weeks and up to 8 weeks of age, and maternal pretreatment with bumetanide did not rescue this deficit (Supplementary Fig. 5a and Table 14**)**. Finally, our results show that maternal pretreatment with bumetanide did not delay the onset of mortality in Mecp2^−/y^ mice (Supplementary Fig. 5b and Table 15**)**. These results confirm that maternal pretreatment with bumetanide does not ameliorate the breathing patterns, weight gain nor onset of mortality of Mecp2^−/y^ mice suggesting that hippocampal GABA signaling is not directly contributing to these deficiencies.

## Discussion

Whether RTT is associated with early alterations - clinically or infraclinically – is clearly a complex and controversial issue. Here, we have performed for the first time recordings immediately after birth and showed that cellular and network developmental patterns are already altered at birth and early post-natal life in Mecp2^−/y^ mice. Indeed, the GABA developmental sequence is affected by this mutation leading to the long-term persistence of GABA excitatory actions. Transient maternal administration of bumetanide around birth induces the recuperation 2 weeks later of inhibitory GABA actions, spontaneous glutamatergic and GABAergic networks activities, and metabotropic LTD similar to age-matched controls. Therefore, already at birth, there are premises of GABA-mediated pathogenic events that might directly or indirectly lead to long-term deleterious sequels. Among these, we evaluated if bumetanide treatment could rescue breathing patterns and weight deficiencies as well as the onset of mortality, and we observed that reducing [Cl^−^]_i_ levels did not attenuate these sequels. RTT is a complex and multifactorial disorder, so it will be interesting to further evaluate other parameters that were not in the scope of our study. Indeed, in recent studies, we showed that bumetanide attenuates the severity of ASD in two animal models and in children with autism^31,32,46^. As RTT includes cognitive ASD-like symptoms, bumetanide treatment in pilot trials might alleviate some of the manifestations present in RTT patients.

Because of the assumption that RTT is not a developmental disease, studies on the roles played by Mecp2 during embryonic and early postnatal development have been largely neglected. Yet, clinical studies have now reported consistent impairments before the expression of overt signs^47^ with one study particularly describing an hemizygote MECP2 male patient displaying a severe pathological condition as early as birth^48^. Using high-resolution chromosome microarray analysis to screen 108 fetuses with congenital structural abnormalities, fetuses with the MECP2 duplication have been identified, presenting ventriculomegaly, hydrocephalus, agenesis of the corpus callosum, choroid plexus cysts, fetal growth restriction and hydronephrosis^49^. In addition, pluripotent stem cells derived from RTT patients or conditionally targeted for Mecp2 display defective structural and functional maturation^50–52^. In experimental studies, the artificial increase of Mecp2 levels in chick embryos and mouse neural progenitor cells can impair neurogenesis and lead to premature neuronal differentiation^53,54^. Moreover, a reduction of neuronal responsiveness to stimuli is reported in neuronal cultures of Mecp2^−/y^ embryonic mice, suggesting, together with previous studies, that Mecp2 could be a potential modulator of prenatal brain development^55^. In keeping with this, invalidating in part or completely Mecp2 impacts very early steps of brain maturation, proliferation, migration and neuronal growth^56–58^. Collectively, these observations suggest that Mecp2 mutations might affect intrauterine development, although this issue deserves direct experimental investigation. However, to the best of our knowledge, only 3 studies have examined early electrical alterations at P14 to P21^45^ and P6 to P23^59,60^. Our present results report alterations at birth suggesting that the intrauterine deficiency generated by the mutation might be attenuated or aggravated by labor and birth. Future investigations are required to validate the former or the latter.

The observation that the oxytocin-mediated neuroprotective action of GABA is abolished in RTT with high [Cl^−^]_i_ present in CA3 pyramidal neurons at birth parallels our earlier studies in the intrauterine VPA and Fragile X rodent models of ASD^31^, and the maternal immune activation (MIA) mouse model that is associated with ASD features^61^. In addition, the developmental GABA shift is also altered in these models leaning to a more depolarized state (also see^34,62^), a pattern of activity similar to the one found in the present study. This result is correlated with the labeling of the neuron-specific chloride exporter KCC2 that is reduced in our Mecp2^−/y^ mice. Interestingly, other studies have shown similar alterations of KCC2 expression and GABA activity dependent on chloride homeostasis^31,62^. In addition, girls with RTT have reduced KCC2 levels in the cerebrospinal fluid^63^ and human neurons derived from pluripotent stem cells from patients with RTT have a significantly reduced KCC2 expression correlated with a delayed GABA switch from an excitatory to an inhibitory action^64^. Moreover, the overexpression of KCC2 rescued the GABA developmental switch deficit in neurons recorded from Mecp2-deficient mice^64^, thus inferring that KCC2 is a critical downstream gene target of MeCP2. Altogether, these results reinforce the observation of an interconnection between [Cl^−^]_i_, GABA activity, Mecp2 deficiency and KCC2 expression, defining the pathogenesis of RTT.

What are the mechanistic links between GABA action and this multiplicity of alterations in RTT? Several observations suggest a link between Mecp2, chloride co-transporters (such as KCC2) and BDNF. Indeed, there are reciprocal neuronal activity-dependent associations of Mecp2 with the BDNF promoter^65^, with Mecp2 transcriptionally regulating BDNF expression^66^. Therefore, it is not surprising to find reduced BDNF levels in Mecp2-deficient mice^67^. BDNF is involved in numerous pathways including the proper maturation of the central respiratory rhythm^68^, and the treatment with a BDNF loop-domain mimetic is known to reverse apneas and respiratory abnormalities in Mecp2-null mice^69^. BDNF also modulates the development of GABAergic neurons and synapses since GABAergic innervation on the soma of BDNF-lacking neurons is reduced^70^, whereas BDNF overexpression improves neurological symptoms in Mecp2-deficient mice^67^ (for a review see^71^). In addition, BDNF controls the inhibitory strength of GABA by modulating both the amount of ion flux through open GABA_A_ channels and [Cl^−^]_i_^72^. Therefore, the GABAergic signals in RTT might be affected because of alterations in BDNF signalization, leading to a loss of the GABA developmental shift with persistent excitatory actions. Future studies should focus on BDNF developmental levels and signalization in combination with bumetanide treatment.

Specific GABAergic signaling alterations have been reported in cortical and brain stem neurons of Mecp2-deficient mice. Thus, GABAergic miniature postsynaptic currents in the ventrolateral medulla of P7 mice are altered due to a reduced release of GABA^59^, while increased tonic currents and extrasynaptic GABA_A_R expression are observed in locus coeruleus neurons of 3 weeks old mice^73^ (also see^74^). Moreover, in Mecp2-mutant mice, the GABA acting agent midazolam alleviates respiratory deficiencies^75^, while phenobarbital rescues dendritic and synaptic defects as well as network activity^76^. Therefore, the critical properties of GABA signaling might be differently impacted in Mecp2^−/y^ mice depending on the structure observed.

In addition to these distinct GABAergic alterations, a wide range of modifications of the glutamatergic/GABAergic balance have been described in adult rodent models of RTT. Indeed, in the cortex and hippocampus, a reduction of the GABAergic inhibitory tone, altered glutamatergic activity and impaired synaptic inhibition have been reported^77–83^. A consistent observation is an enhanced tendency to hypersynchronous activity^39^ that together with the previously described alterations underlies the relatively high incidence of epilepsies in girls with RTT like in animal models^77,84,85^. Interestingly, depending on the type of GABAergic interneurons presenting a loss of Mecp2, different RTT features are induced. Thus, selective invalidation of Mecp2 in parvalbumin-containing interneurons abolishes the critical period plasticity^86^ whereas its invalidation in somatostatin interneurons leads to stereotyped behavior and seizures^87^. However, all these studies are performed in adult neurons and therefore not relevant to the issue of developmental alterations in RTT. Yet, determining early pre-clinical changes is important in a cognitive and therapeutic perspective.

Finally, we can hypothesize a direct link in the mechanistic relationship between GABA actions and the early phase of mGluR-dependent LTD. Indeed, three studies have reported that the level of inhibition modulates the amplitude of mGluR-LTD in the hippocampus. Interestingly, in two of them, GABA_A_-receptor-mediated inhibition enhanced mGluR-LTD^88,89^, and in the third one, blocking GABA_A_-receptor-mediated inhibition abolished low frequency stimulation-induced mGluR LTD^90^. Also, other studies have linked chloride homeostasis with glutamatergic synaptic plasticity. Indeed, Deidda *et al*.^91^ reported that high frequency stimulation-induced LTP is reduced in Ts65Dn mice (a mouse model of Down syndrome in which GABA remains depolarizing) and bumetanide bath application completely rescued it. In addition, Pavlov *et al*.^92^ reported that a GABA_A_-receptor-mediated depolarization is required for the induction of hippocampal NMDA-dependent LTD. We cannot exclude the possibility that persistent depolarizing actions of GABA may have affected the developmental sequence, expression or activity of factors contributing to mGluR-LTD, such as calcium conductance, glutamate receptors expression or kinases/phosphatases actions. Also, because the excitation/inhibition ratio is altered in Mecp2-null mice, it is possible that this alteration in ongoing synaptic activity affects the mGluR-LTD.

Maternal administration of bumetanide around birth restored the inhibitory action of GABA 2 weeks later. As the life time of bumetanide is short in infants^93^, a persistence of the agent in offspring can be excluded, thus suggesting that the restoration of low [Cl^−^]_i_ during a brief period impacts the action of GABA weeks after birth. Maternal bumetanide also attenuated the LTD deficit in slices recorded weeks later, again suggesting long-term effects of interventions during labor and birth. To the best of our knowledge, this is the first evidence that the polarity of GABA at birth can impact synaptic plasticity weeks later. It is difficult at this stage of our knowledge of these long-term effects of bumetanide to speculate on their underlying mechanisms as they could be due to a large number of possibilities including permanent alterations of pre or post- synaptic mechanisms. Indeed, [Cl^−^]_i_ plays a crucial role on the trophic action that GABA exerts on all developmental mechanisms from proliferation to differentiation, growth, and synapse formation^23,94^. Nevertheless, it is manifest that the critical period of birth impacts the GABA developmental sequence and of synaptic plasticity.

Our results complement similar observations in other pathological conditions. Thus, maternal pretreatment with bumetanide attenuated persistently electrical and behavioral features of ASD in the intrauterine VPA and the Fragile X rodent models^31,32^, reinforcing the suggestion that short-term treatment with bumetanide induces long-term beneficial effects. Early oxytocin (OT) administration also attenuated the long-term sequels of neonatal maternal separation, notably pain sensitivity that is restored in adults^95^. OT and bumetanide exert analgesic actions on pups by reducing [Cl^−^]_i_ and restoring GABAergic inhibition in pain pathways^96^. Similarly, postnatal administration of OT during early life in both babies and mice deficient for Magel2 prevents feeding deficiency in pups as well as social and memory deficits in adults^97–100^. It is suggested that GABAergic and glutamatergic activities during labor and birth exert long-term sequels by impacting essential early sensory interactions between the mother and pups, reinforcing the idea that labor and birth are critical periods^23^. Interestingly, birth itself acts as an active trigger to accelerate circuit formation of the barrel cortex and suction^101,102^. Given that the loss of Mecp2 results to a dysregulation of a large number of pathways already at birth, early interventions might prevent the vicious cycle triggered by early insults. Bumetanide however failed to correct several general clinical parameters including breathing deficiency and weight gain as well as the onset of mortality in Mecp2^−/y^ offspring, suggesting that the polarity of GABA at birth is not involved in these mechanisms. This also raises the possibility that some of the pathogenic events have a different developmental sequence occurring after birth consistently with the observation that respiration and weight gain are normal at first then present a delayed regression^45^. Determining whether bumetanide attenuates some of the functionalities impacted in RTT will require additional experiments testing a plethora of clinical and behavioral signs.

In conclusion, results suggest that birth is a critical period in the pathogenesis of Mecp2^−/y^ mice. Developmental sequences are of clear importance in the pathogenesis of RTT and must be investigated as early as birth. Studies on the bidirectional links between intrauterine development, labor and birth are of paramount importance. Unfortunately, our understanding of these events is limited by the paucity of experiments centered on this critical period. A convergence of experimental observations stresses the importance of the delivery-associated GABA developmental shift and the long-term consequences of its abolition by genetic or environmental intrauterine insults. These experimental observations are reinforced by the clinical efficacy of bumetanide to treat ASD. Although we do not know how administration of a drug restricted to the critical period of birth leads to such long-term effects, understanding the developmental alterations associated with this period is instrumental in a therapeutic perspective.

## Material and Methods

### Mice

Experiments were performed on Mecp2-null (Mecp2^−/y^) and wt (Mecp2^+/y^) littermate mice maintained on a C57BL/6 background^6^. All experiments were conducted in accordance with the European Community Council Directives (2010/63/UE) and approved by the local ethics committee (CEEA n°14) and French Ministry for Research (Project #16370-2018073116436588). Postnatal mice and litters were randomly assigned for recordings except for the cohort of the LTD experiments.

### Slice preparation

Mecp2^−/y^ and wt littermates (P0, P14–P16) were euthanized by decapitation. The brain was rapidly removed and placed in an oxygenated ice-cold choline solution containing (in mM): 132.5 choline chloride, 2.5 KCl, 0.7 CaCl_2_, 3 MgCl_2_, 1.2 NaH_2_PO_4_, 25 NaHCO_3_ and 8 glucose; oxygenated with 95% O_2_ and 5% of CO_2_. Transverse 300–400 μm-thick horizontal slices were cut using a vibratome (Leica VT1000S; Leica Microsystems, Germany) in ice-cold choline solution oxygenated with 95% O_2_ and 5% of CO_2_. Before recording, slices were incubated in an artificial cerebrospinal fluid (ACSF) solution (see compositions below) equilibrated at pH 7.4 with 95% O_2_ and 5% CO_2_ at room temperature (22–25 °C) for at least 1 h to allow recovery. For the recordings, slices were placed into the recording chamber where they were fully submerged and superfused with oxygenated ACSF solution at room temperature (22–25 °C) and at 34 °C for LTD experiments. For the recordings of glutamatergic and GABAergic spontaneous activities, we used ACSF with the following composition (in mM): 125 NaCl, 3.5 KCl, 2 CaCl_2_, 1 MgCl_2_, 1.25 NaH_2_PO_4_, 26 NaHCO_3_ and 10 glucose. For single-channel, extracellular field potential, miniature postsynaptic currents (mPSCs) and LTD, recordings we used ACSF containing (in mM): 126 NaCl, 3.5 KCl, 2 CaCl_2_, 1.3 MgCl_2_, 1.2 NaH_2_PO_4_, 25 NaHCO_3_ and 11 glucose.

### Electrophysiological recordings

#### Cell-attached recordings

Cell-attached recordings of action potentials firing were performed in CA3 pyramidal cells at Vp = 0 mV with an EPC-10 amplifier (HEKA Elektronik Dr Schulze GmbH, Germany) using 8–10 MΩ borosilicate glass pipettes filled with ACSF. Isoguvacine (10 µM) was applied locally with a microfluidic pipette for the time necessary to reach saturation of isoguvacine effects. Recordings of single GABA_A_ receptor channels in cell-attached configuration were performed in CA3 pyramidal cells using an EPC-10 amplifier (HEKA Elektronik Dr Schulze GmbH, Germany). Patch pipette solution for recordings of single GABA_A_ channels contained (in mM): 120 NaCl, 5 KCl, 20 TEA-Cl, 5 4-aminopyridine, 0.1 CaCl_2_, 10 MgCl_2_, 10 glucose, 10 HEPES-NaOH buffered to pH 7.3 and GABA (5 μM). Pipettes had a resistance in the range of 7–10 MΩ. We performed conventional cell-attached recordings of currents through GABA_A_ channels after gigaseal formation (>3 GΩ). Currents through GABA_A_ channels were recorded from −100 to +60 mV with 10 mV increments for 1–2 minutes for each holding potential, depending on the ongoing frequency of GABA_A_ channels openings, to obtain at least 20 single-channel openings for each potential. Bumetanide (acute, 10 µM) was added in the bath solution for 15 minutes before single-channels recordings. Action potential frequency and single-channels recordings were analyzed using Clampfit 10.4 (Molecular Devices, CA, USA) and OriginPro (OriginLab Corporation, MA, USA) softwares. The exclusion criteria for recordings were insufficient signal-to-noise ratio, insufficient number of channel openings, presence of strong irregular artefact signals, strong baseline oscillations, short dwell time of channels, too fast desensitization of channels or presence of non-specific conductances. We also excluded sublevel channel openings using threshold detection.

#### Whole-cell recordings

Whole-cell recordings of CA3 pyramidal neurons were performed using an EPC-10 amplifier (HEKA Elektronik Dr Schulze GmbH, Germany). Patch pipette solution contained (in mM): 130 K-gluconate, 10 Na-gluconate, 7 NaCl, 4 Mg-ATP, 10 HEPES, 4 phosphocreatine, 0.3 Na-GTP, pH 7.3 with KOH. Spontaneous GABAergic postsynaptic currents (sIPSC) were recorded for 15 min at the reversal potential for glutamatergic currents (+10 mV), and spontaneous glutamatergic postsynaptic currents (sEPSC) were recorded for 10 min at the reversal potential for GABAergic currents (−75 mV). We defined as a burst a minimum of three sEPSC associated with a baseline elevation of the amplitude of minimum 60 pA. Bumetanide (acute, 10 µM) was added in the bath solution for 20 minutes before sEPSC or sIPSC recordings.

Miniature postsynaptic currents (mPSCs) were recorded in whole-cell voltage-clamp configuration with a MultiClamp 700B amplifier (Molecular Devices, CA, USA). To record miniature excitatory postsynaptic currents (mEPSC), micropipettes (6–8 MΩ) were filled with the following recording solution (in mM): 130 K-gluconate, 10 Na-Gluconate, 4 NaCl, 10 HEPES, 4 phosphocreatine, 4 Mg-ATP and 0.3 Na-GTP and recordings were performed at −70 mV in the presence of TTX (1 μM) and gabazine (5 μM). To record miniature inhibitory postsynaptic currents (mIPSC), micropipettes (4–6 MΩ) were filled with the following recording solution (in mM): 130 KCl, 10 HEPES, 1.1 ethylene glycol-bis (β-aminoethyl ether)-N,N,N′,N′-tetra-acetic acid (EGTA), 0.1 CaCl_2_, 5 phosphocreatine, 4 Mg-ATP and 0.4 Na-GTP and recordings were performed at −70 mV in the presence of TTX (1 μM) and CNQX (10 μM). All following parameters were controlled to be within acceptable and similar range across recordings: series resistance, membrane capacitance, resting membrane potential (i.e. membrane potential when I = 0, Vrest), firing pattern and holding current.

Cells with holding currents >−30 pA (for P0) and >−40 to −50 pA (for P15) at holding potential −70 mV were excluded from analysis. Holding current and input resistance were constantly monitored and cells with >15% shift of these parameters during the recordings were excluded from analysis.

Spontaneous and miniature IPSC and EPSC were analyzed using Mini Analysis 6.0.7 (Synaptosoft, GA, USA) and OriginPro (OriginLab Corporation, MA, USA) softwares.

For MiniAnalysis the threshold for events detection was defined as 3 times the standard deviation of the noise. The exclusion criteria for recordings were insufficient signal-to-noise ratio, baseline oscillations and distorted shape of synaptic events. mIPSC and mEPSC were analyzed by an investigator blind to the genotype.

#### Extracellular recordings

Extracellular field potential recordings were performed in the CA3 pyramidal layer of Mecp2^−/y^ and wt littermates using glass pipettes (Harvard Apparatus, MA, USA) containing ACSF. Extracellular field potentials were recorded with a DAM-80A amplifier (WPI, UK) with a low-pass filter at 1 Hz and high-pass filter at 3 kHz. Data were digitized online with a Digidata 1400A digitizer (Molecular Devices, CA, USA) and analyzed using Clampfit 10.4 software (Molecular Devices, CA, USA). Isoguvacine (10 µM) was directly added to the perfusion solution for 90 s. The threshold for spike detection was defined as 3 times the standard deviation of the noise recorded in the bath solution. We calculated the spike frequency for control, isoguvacine and wash-out periods, and if the latter did not come back to control levels (±20% of control), the slice was excluded from the analysis.

For LTD experiments, a bipolar stimulating electrode made from twisted nichrome wire (66 μm; A-M Systems, WA, USA) was placed on the surface of the stratum radiatum of CA1 to stimulate the Schaffer collaterals/commissural fibers (10–50 µs, 5–15 V, 0.03 Hz). Extracellular 50 µm tungsten electrodes (California Fine Wire, CA, USA) were used to record dendritic field excitatory postsynaptic potentials (fEPSP) from the stratum radiatum of the CA1 region. The signals were amplified using a DAM80 amplifier (WPI, UK), digitized with an Axon Digidata 1550B (Molecular Devices, CA, USA), recorded with Axoscope software version 8.1 (Molecular Devices, CA, USA) and analyzed offline with Mini Analysis Program version 6.0 (Synaptosoft, GA, USA) by measuring the onset (a 30–70% rising phase) slope of the fEPSP. LTD was induced with (*S*)-3,5-Dihydroxyphenylglycine (DHPG, 50 μM for 5 min). The inclusion criteria of slices consisted on a stable and high quality basal synaptic response for at least 20 minutes after stimulation and before applying the DHPG protocol.

### Immunohistochemistry

At P15, wt and Mecp2^−/y^ mice were perfused intracardially with antigenfix (Diapath, Italy) and brains were sliced (70 µm thickness) using a Leica VT1000S vibratome (Leica Microsystems, Germany). Selected sections from wt and Mecp2^−/y^ hippocampi were processed in parallel for immunohistochemistry under identical conditions. Sections were incubated for 1 h at room temperature, with a mixture of 5% normal goat serum (NGS, Jackson ImmunoResearch Laboratories, Inc., PA, USA) in 0.1 M phosphate buffered saline (PBS, Life Technologies, CA, USA) with 0.3% Triton X-100 (Sigma-Aldrich, MO, USA). They were then incubated overnight at 4 °C with the anti-KCC2 antibody (rabbit; 1/800; US Biological, MA, USA) diluted in PBS with NGS 1% and 0.1% Triton X-100. After being washed in PBS, sections were incubated for 1 h at room temperature with the fluorescent-labeled secondary antibody Alexa Fluor 555 (1/1000, Life Technologies, CA, USA) diluted in PBS. Sections were finally mounted on slides and imaged on a SP5X Leica confocal microscope (Leica Microsystems, Germany) using identical settings (2 hippocampal hemispheres of 3 sections per animal). KCC2 immunofluorescence analysis of the whole CA3 pyramidal layer within the image was performed blindly using the open-source platform Fiji (1.50e, Java 1.8.0_60, 64-bit; https://fiji.sc/)^103^.

### Plethysmography experiments

3 groups of mice were used at different ages in this study: wt, Mecp2^−/y^ and Mecp2^−/y^ pretreated with bumetanide (see the Supplementary Table 13).

#### Plethysmographic recording

Breathing of unrestrained, non-anesthetized mice was recorded using constant air flow whole-body plethysmography filled with air (EMKA Technologies, France) at different ages for each mouse (from 3.5 to 8 weeks old). Four plethysmography chambers of 200 mL containing air (calibrated by injecting 1 mL of air) maintained at 25 ± 0.5 °C were used to allow simultaneous measurements. Breathing cycles were recorded for 1 h under normal-ventilation conditions after an adaptation phase of 1 h in the experimental room and 44 min in the recording chamber. All animals evaluated for respiration were included in the analysis.

#### Analysis

Analog signals were obtained using an usbAMP device equipped with four inputs and processed using EMKA technologies IOX software (EMKA Technologies, France). Data analyses were carried out using MatLab software (MathWorks, MA, USA).

One breathing cycle consisted of a period of inspiration followed by a period of expiration. Plethysmography signals were converted into EDF files and analyzed using custom-developed analysis software in MatLab (MathWorks, MA, USA). For each animal, analysis was conducted on the time window of 1 h following the animal adaptation. In order to identify the positive and negative parts of a breathing cycle, the signal s(t) was first pre-processed through double thresholding and transformed into a trinary time series b(t) according to the following (where t is the time frame):

b(t) = 1 for s(t) > 0.15

b(t) = −1 for s(t) < −0.4

b(t) = 0 for −0.4 ≤ s(t) ≤ 0.15.

Next, a moving average over 25 frames was applied to b(t) to smooth over local fluctuations and again converted into a trinary time series by setting positive (negative) values to one (minus one). This entire pre-processing procedure allowed to reliably identify the positive and negative parts of signals corresponding to the two phases of the breathing cycles (inspiration and expiration respectively). The beginning of positive epochs, characterized by consecutive positive values in the time series, was used as the starting timing of the positive cycles. Similar analysis was done to identify the starting timing of the negative cycles. The analysis described in this paper refers to the interval between consecutive positive cycles with at least a negative cycle in between (inter-breathing cycle-interval, ICI). However, no statistical difference was observed when we considered the opposite case, i.e. the interval between negative cycles.

For each animal, the cumulative probability of the ICI was calculated (shown in Supplementary Fig. 4b). The matrix displaying the distance between the ICI datasets from each pair of animals was calculated using the Cliff’s Delta effect size coefficient (Supplementary Fig. 4c)^104^. Hierarchical clustering analysis was performed on the Cliff’s Delta matrix using a cosine distance (as depicted in Supplementary Fig. 4d) and was aimed at identifying three groups in the dataset. The statistical significance of the clustering result was assessed in relation to the probability distribution obtained in random classifications which is given by a binomial distribution with three possible choices and a number of repetitions equal to the number of animals per group. Concerning the statistical power, the p-values calculated from the binomial distribution for the number of animals classified in each group (as shown in Supplementary Fig. 4d) were lower than 10^−18^.

### Weight gain and life span

Wt, Mecp2^−/y^ and Mecp2^−/y^ mice pretreated with bumetanide were weighted weekly from 3.5 to 8 weeks of age, and the day of death was noted as the onset of mortality. Wt mice were evaluated for the onset of mortality until 30–38 weeks of age. All animals randomly assigned for these tests were analyzed by an investigator blind to the treatment and genotype.

### Pharmacological agents and bumetanide pretreatment

*In vitro*. Bumetanide (10 μM, Sigma-Aldrich, MO, USA), isoguvacine (10 μM, Sigma-Aldrich, MO, USA), TTX (1 μM, Abcam, Bristol, UK), CNQX (10 μM, Sigma-Aldrich, MO, USA), gabazine (5 μM, Tocris Cookson, Bristol, UK) and DHPG (50 μM, Tocris Bioscience, UK) were directly added to the perfusion solutions.

*In vivo*. Pregnant mice were randomly assigned for bumetanide pretreatment (2.5 mg/kg) administered in the drinking water.

### Statistical analysis

Datasets were first tested for normality. If the datasets fit a normal distribution, a parametric method was used, and if not, a nonparametric method was used. Electrophysiological studies were analyzed with the two-tailed t-test, paired sample two-tailed t-test, Mann-Whitney test, two-tailed Fisher’s exact test with Bonferroni correction, one-way analysis of variance (ANOVA) with Fisher’s least significant difference (LSD) or Tukey’s post-hoc tests, repeated measures ANOVA with Tukey’s post-hoc test, and Kruskal–Wallis with Dunn’s multiple comparison post-hoc test (see Supplementary material for descriptive statistics). KCC2 immunofluorescence was analyzed with the Mann-Whitney test. Plethysmography studies were analyzed as described in the previous section. Weight gain and life span were analyzed with one-way ANOVA with Bonferroni post-hoc test. Analyzes were performed with GraphPad Prism 7 (GraphPad Software, CA, USA) or OriginPro (OriginLab Corporation, MA, USA). Statistical power was analyzed using AnaStats tools (Anastats Scop ARL, France). All data are presented as mean ± SEM. *p < 0.05; **p < 0.01; ***p < 0.001.

## Supplementary information

Supplementary materials

## Data Availability

The datasets generated and analyzed during the current study are available from the corresponding author on reasonable request.
