# Supplementary material for: Early alterations in a mouse model of Rett syndrome: the GABA developmental shift is abolished at birth
Source: Sci Rep. 2019 Jun 25;9:9276. doi: 10.1038/s41598-019-45635-9 (PMC6592949; doi:10.1038/s41598-019-45635-9)

Supplementary materials

Early alterations in a mouse model of Rett syndrome: the GABA developmental shift is abolished at birth

Lozovaya N[1], Nardou R[1], Tyzio R[1, 2], Chiesa M[1, 2], Pons-Bennaceur A[2], Eftekhari S[1, 2], Bui T[1, 2], Billon-Grand M[1], Rasero J[3], Bonifazi P[3, 4], Guimond D[1], Gaiarsa J-L[2], Ferrari DC[1], and Ben-Ari Y[1*].

[1] Neurochlore, Ben-Ari Institute of Neuroarcheology, Bâtiment Beret-Delaage, Parc scientifique et technologique de Luminy, 13288 Marseille cedex 09, France.

[2] Mediterranean Institute of Neurobiology (INMED), Department of Neurobiology, Aix-Marseille University, INSERM U1249, Marseille, France.

[3] Biocruces Health Research Institute, 48903 Barakaldo, Spain

[4] IKERBASQUE: The Basque Foundation for Science, 48013 Bilbao, Spain

* Address for correspondence: ben-ari@neurochlore.fr

Key words: GABA/glutamate imbalance, neonatal neuronal activity, bumetanide, breathing patterns

Supplementary Tables

Statistical analysis. Significant differences are highlighted in blue.

Supplementary Table 1

Figure 1a, b
DF_{GABA} (P0) in wt, Mecp2$^{-/y}$ and Mecp2$^{-/y}$ mice with acute treatment of bumetanide (acute).
Two-tailed t-test.

Groups	n (cells)	N (mice)	Mean ± SEM (mV)	Two-tailed t-test
wt	15	3	-1.28 ± 3.69	0.003
Mecp2$^{-/y}$	28	3	10.35 ± 1.84	
Mecp2$^{-/y}$	22	3	9.47 ± 1.46	$7*10^{-8}$
Mecp2$^{-/y}$ bumetanide (acute)	20	3	-9.41 ± 2.54	

Power

Groups	Alpha	Power
wt vs Mecp2$^{-/y}$	0.05	0.89
Mecp2$^{-/y}$ vs Mecp2$^{-/y}$ bumetanide (acute)	0.05	1

Figure 1d
DF_{GABA} (P15) in wt and Mecp2$^{-/y}$ mice.
Two-tailed t-test.

Groups	n (cells)	N (mice)	Mean ± SEM (mV)	Two-tailed t-test
wt	17	3	1.47 ± 2.52	0.01
Mecp2$^{-/y}$	17	3	9.50 ± 1.56	

Power

Groups	Alpha	Power
wt vs Mecp2$^{-/y}$	0.05	0.77

Supplementary Figure 1b
KCC2 immunofluorescence (P15) in wt and Mecp2$^{-/y}$ mice.
Mann-Whitney test.

Groups	N (mice)	Mean ± SEM (normalized to control)	Mann-Whitney test
wt	7	1.00 ± 0.03	0.0007
Mecp2$^{-/y}$	9	0.79 ± 0.03	

Power

Groups	Alpha	Power
wt vs Mecp2$^{-/y}$	0.05	0.95

Supplementary Table 2

Figure 2e

Effects of isoguvacine on spontaneous spiking frequency (cell-attached recordings) normalized to control in CA3 hippocampal pyramidal neurons (P15) in wt, $Mecp2^{-/y}$ and bumetanide-pretreated (MP) $Mecp2^{-/y}$ mice.

Paired sample two-tailed t-test intragroup and one-way ANOVA with Fisher's LSD post-hoc test.

Groups	n (cells)	N (mice)	Mean ± SEM (normalized to baseline frequency)	Paired sample two-tailed t-test
wt	6	3	0.30 ± 0.08	$3*10^{-4}$
$Mecp2^{-/y}$	11	7	2.42 ± 0.25	$2*10^{-4}$
$Mecp2^{-/y}$ bumetanide (MP)	10	3	0.81 ± 0.05	0.009

Groups	n (cells)	n (cells)	Univariate ANOVA between groups	Fisher's LSD post-hoc test
wt vs $Mecp2^{-/y}$	6	11	$5*10^{-8}$	$8*10^{-8}$
$Mecp2^{-/y}$ bumetanide (MP) vs wt	10	6		0.08
$Mecp2^{-/y}$ bumetanide (MP) vs $Mecp2^{-/y}$	10	11		$6*10^{-7}$

Power

Groups	Alpha	Power
wt vs $Mecp2^{-/y}$ vs $Mecp2^{-/y}$ bumetanide (MP)	0.05	0.99

Supplementary Table 3

Figure 3b
Effects of isoguvacine on spontaneous activity (spontaneous extracellular field potentials) normalized to control (P15) in wt, Mecp2$^{-/y}$ and bumetanide-pretreated (MP) Mecp2$^{-/y}$ mice.
Repeated measures ANOVA with Tukey's post-hoc test intragroup and one-way ANOVA with Tukey's post-hoc test.

Groups	n (slices)	N (mice)	Mean ± SEM (% of control)
wt isoguvacine	20	3	77.30 ± 2.80
wt washout	20	3	99.70 ± 2.70
Mecp2$^{-/y}$	21	3	99.42 ± 3.73
Mecp2 washout	21	3	102.5 ± 2.51
Mecp2$^{-/y}$ bumetanide (MP)	16	5	80.90 ± 5.05
Mecp2 bumetanide washout	16	5	98.36 ± 3.56

Group wt	Repeated measures ANOVA	Tukey's post-hoc test
Control vs isoguvacine	<0,0001	<0,0001
Isoguvacine vs washout		0.9933
Control vs washout		<0,0001

Groups Mecp2$^{-/y}$	Repeated measures ANOVA	Tukey's post-hoc test
Control vs isoguvacine	0.5996	0.9867
Isoguvacine vs washout		0.5793
Control vs washout		0.7299

Group Mecp2$^{-/y}$ bumetanide (MP)	Repeated measures ANOVA	Tukey's post-hoc test
Control vs isoguvacine	0.0035	0.0048
Isoguvacine vs washout		0.8902
Control vs washout		0.0352

Groups	Univariate ANOVA between groups	Tukey's post-hoc test
wt vs Mecp2$^{-/y}$	0.0002	0.0003
wt vs Mecp2$^{-/y}$ bumetanide (MP)		0.7979
Mecp2$^{-/y}$ vs Mecp2$^{-/y}$ bumetanide (MP)		0.0043

Power

Groups	Alpha	Power
Group wt	0.05	1
Group Mecp2$^{-/y}$	0.05	0.11
Group Mecp2$^{-/y}$ bumetanide (MP)	0.05	0.91
wt vs Mecp2$^{-/y}$ vs Mecp2$^{-/y}$ bumetanide (MP)	0.05	0.94

Supplementary Table 4

Figure 4c
sEPSC frequency at P0 in wt and Mecp2$^{-/y}$ mice.
Mann-Whitney test.

Groups	n (cells)	N (mice)	Mean ± SEM (Hz)	Mann-Whitney test
wt	17	4	0.69 ± 0.14	0.0003
Mecp2$^{-/y}$	24	10	3.01 ± 0.68	

Power

Groups	Alpha	Power
wt vs Mecp2$^{-/y}$	0.05	0.79

Figure 4d
sEPSC charge density at P0 in wt and Mecp2$^{-/y}$ mice.
Mann-Whitney test.

Groups	n (cells)	N (mice)	Mean ± SEM (normalized to control)	Mann-Whitney test
wt	16	4	1.00 ± 0.31	0.001
Mecp2$^{-/y}$	22	10	6.99 ± 1.56	

Power

Groups	Alpha	Power
wt vs Mecp2$^{-/y}$	0.05	0.86

Figure 4e
sEPSC amplitude at P0 in wt and Mecp2$^{-/y}$ mice.
Mann-Whitney test.

Groups	n (cells)	N (mice)	Mean ± SEM (pA)	Mann-Whitney test
wt	17	4	4.61 ± 0.45	0.0456
Mecp2$^{-/y}$	24	10	5.32 ± 0.35	

Power

Groups	Alpha	Power
wt vs Mecp2$^{-/y}$	0.05	0.21

Supplementary Table 5

Figure 4f
sIPSC frequency at P0 in wt and Mecp2$^{-/y}$ mice.
Mann-Whitney test.

Groups	n (cells)	N (mice)	Mean ± SEM (Hz)	Mann-Whitney test
wt	13	3	3.29 ± 0.77	0.65
Mecp2$^{-/y}$	15	7	2.86 ± 0.67	

Power

Groups	Alpha	Power
wt vs Mecp2$^{-/y}$	0.05	<10%

Figure 4g
sIPSC charge density at P0 in wt and Mecp2$^{-/y}$ mice.
Mann-Whitney test.

Groups	n (cells)	N (mice)	Mean ± SEM (normalized to control)	Mann-Whitney test
wt	12	3	1.00 ± 0.22	0.82
Mecp2$^{-/y}$	15	7	1.07 ± 0.20	

Power

Groups	Alpha	Power
wt vs Mecp2$^{-/y}$	0.05	<10%

Figure 4h
sIPSC amplitude at P0 in wt and Mecp2$^{-/y}$ mice.
Mann-Whitney test.

Groups	n (cells)	N (mice)	Mean ± SEM (pA)	Mann-Whitney test
wt	17	4	14.36 ± 1.22	0.61
Mecp2$^{-/y}$	24	10	14.12 ± 0.99	

Power

Groups	Alpha	Power
wt vs Mecp2$^{-/y}$	0.05	<10%

Supplementary Table 6

Figure 5c
sEPSC frequency at P15 in wt, Mecp2$^{-/y}$ and bumetanide-pretreated (MP) Mecp2$^{-/y}$ mice.
Kruskal-Wallis with Dunn's multiple comparison post-hoc test.

Groups	n (cells)	N (mice)	Mean ± SEM (Hz)
wt	19	3	7.29 ± 0.86
Mecp2$^{-/y}$	28	7	20.12 ± 2.87
Mecp2$^{-/y}$ bumetanide (MP)	18	4	5.82 ± 0.55

Groups	n (cells)	n (cells)	Kruskal-Wallis test	Dunn's multiple comparison post-hoc test
wt vs Mecp2$^{-/y}$	19	28	<0.0001	0.0015
Mecp2$^{-/y}$ bumetanide (MP) vs wt	18	19		0.72
Mecp2$^{-/y}$ bumetanide (MP) vs Mecp2$^{-/y}$	18	28		0.0001

Power

Groups	Alpha	Power
wt vs Mecp2$^{-/y}$ vs Mecp2$^{-/y}$ bumetanide (MP)	0.05	0.95

Figure 5d
sEPSC charge density at P15 in wt, Mecp2$^{-/y}$ and bumetanide-pretreated (MP) Mecp2$^{-/y}$ mice.
Kruskal-Wallis test with Dunn's multiple comparison post-hoc test.

Groups	n (cells)	N (mice)	Mean ± SEM (normalized to control)
wt	20	3	1.00 ± 0.19
Mecp2$^{-/y}$	28	6	2.53 ± 0.39
Mecp2$^{-/y}$ bumetanide (MP)	18	4	0.99 ± 0.17

Groups	n (cells)	n (cells)	Kruskal-Wallis test	Dunn's multiple comparison post-hoc test
wt vs Mecp2$^{-/y}$	20	28	0.0013	0.0048
Mecp2$^{-/y}$ bumetanide (MP) vs wt	18	20		0.999
Mecp2$^{-/y}$ bumetanide (MP) vs Mecp2$^{-/y}$	18	28		0.0096

Power

Groups	Alpha	Power
wt vs Mecp2$^{-/y}$ vs Mecp2$^{-/y}$ bumetanide (MP)	0.05	0.84

Figure 5g
sEPSC amplitude at P15 in wt, Mecp2$^{-/y}$ and bumetanide-pretreated (MP) Mecp2$^{-/y}$ mice.
Kruskal-Wallis with Dunn's multiple comparison post-hoc test.

Groups	n (cells)	N (mice)	Mean ± SEM (pA)
wt	20	3	9.35 ± 0.94
Mecp2$^{-/y}$	28	6	11.20 ± 0.98
Mecp2$^{-/y}$ bumetanide (MP)	18	4	8.72 ± 0.67

Groups	n (cells)	n (cells)	Kruskal-Wallis test	Dunn's multiple comparison post-hoc test
wt vs Mecp2$^{-/y}$	20	28	0.33	ns
Mecp2$^{-/y}$ bumetanide (MP) vs wt	18	20		ns
Mecp2$^{-/y}$ bumetanide (MP) vs Mecp2$^{-/y}$	18	28		ns

Power

Groups	Alpha	Power
wt vs Mecp2$^{-/y}$ vs Mecp2$^{-/y}$ bumetanide (MP)	0.05	0.31

Supplementary Table 7

Figure 5e
sIPSC frequency at P15 in wt, Mecp2$^{-/y}$ and bumetanide-pretreated (MP) Mecp2$^{-/y}$ mice.
Kruskal-Wallis test with Dunn's multiple comparison post-hoc test.

Groups	n (cells)	N (mice)	Mean ± SEM (Hz)
wt	14	3	14.43 ± 2.18
Mecp2$^{-/y}$	16	6	6.51 ± 1.11
Mecp2$^{-/y}$ bumetanide (MP)	14	4	11.74 ± 1.38

Groups	n (cells)	n (cells)	Kruskal-Wallis	Dunn's multiple comparison post-hoc test
wt vs Mecp2$^{-/y}$	14	16	0.0026	0.0037
Mecp2$^{-/y}$ bumetanide (MP) vs wt	14	14		0.9999
Mecp2$^{-/y}$ bumetanide (MP) vs Mecp2$^{-/y}$	14	16		0.0299

Power

Groups	Alpha	Power
wt vs Mecp2$^{-/y}$ vs Mecp2$^{-/y}$ bumetanide (MP)	0.05	0.81

Figure 5f
sIPSC charge density at P15 in wt, Mecp2$^{-/y}$ and bumetanide-pretreated (MP) Mecp2$^{-/y}$ mice.
Kruskal-Wallis test with Dunn's multiple comparison post-hoc test.

Groups	n (cells)	N (mice)	Mean ± SEM (normalized to control)
wt	14	3	1.00 ± 0.21
Mecp2$^{-/y}$	16	6	0.29 ± 0.05
Mecp2$^{-/y}$ bumetanide (MP)	15	4	0.78 ± 0.11

Groups	n (cells)	n (cells)	Kruskal-Wallis	Dunn's multiple comparison post-hoc test
wt vs Mecp2$^{-/y}$	14	16	0.0003	0.001
Mecp2$^{-/y}$ bumetanide (MP) vs wt	15	14		0.9999
Mecp2$^{-/y}$ bumetanide (MP) vs Mecp2$^{-/y}$	15	16		0.0029

Power

Groups	Alpha	Power
wt vs $Mecp2^{-/y}$ vs $Mecp2^{-/y}$ bumetanide (MP)	0.05	0.81

Figure 5h
sIPSC amplitude at P15 in wt, $Mecp2^{-/y}$ and bumetanide-pretreated (MP) $Mecp2^{-/y}$ mice.
Kruskal-Wallis test with Dunn's multiple comparison post-hoc test.

Groups	n (cells)	N (mice)	Mean ± SEM (pA)
wt	14	3	22.95 ± 2.30
$Mecp2^{-/y}$	15	6	15.81 ± 0.56
$Mecp2^{-/y}$ bumetanide (MP)	15	4	20.73 ± 1.29

Groups	n (cells)	n (cells)	Kruskal-Wallis test	Dunn's multiple comparison post-hoc test
wt vs $Mecp2^{-/y}$	14	16	0.0016	0.0048
$Mecp2^{-/y}$ bumetanide (MP) vs wt	15	14		0.9999
$Mecp2^{-/y}$ bumetanide (MP) vs $Mecp2^{-/y}$	15	16		0.0074

Power

Groups	Alpha	Power
wt vs $Mecp2^{-/y}$ vs $Mecp2^{-/y}$ bumetanide (MP)	0.05	0.73

Supplementary Table 8

Figure 5i
sIPSC/sEPSC ratio in wt, Mecp2$^{-/y}$ and bumetanide-pretreated (MP) Mecp2$^{-/y}$ mice.
Kruskal-Wallis test with Dunn's multiple comparison post-hoc test.

Groups	n (cells)	N (mice)	Mean ± SEM
wt	14	3	14.06 ± 5.45
Mecp2$^{-/y}$	14	6	1.39 ± 0.34
Mecp2$^{-/y}$ bumetanide (MP)	13	4	6.87 ± 1.59

Groups	n (cells)	n (cells)	Kruskal-Wallis test	Dunn's multiple comparison post-hoc test
wt vs Mecp2$^{-/y}$	14	14	<0,0001	0.0002
Mecp2$^{-/y}$ bumetanide (MP) vs wt	13	14		0.9999
Mecp2$^{-/y}$ bumetanide (MP) vs Mecp2$^{-/y}$	13	14		0.0013

Power

Groups	Alpha	Power
wt vs Mecp2$^{-/y}$ vs Mecp2$^{-/y}$ bumetanide (MP)	0.05	0.51

Supplementary Table 9

Supplementary Figure 2b
mEPSC frequency at P15 in wt and Mecp2$^{-/y}$ mice.
Mann-Whitney test.

Groups	n (cells)	N (mice)	Mean ± SEM (Hz)	Mann-Whitney test
wt	12	5	0.67 ± 0.12	0.59
Mecp2$^{-/y}$	19	6	0.77 ± 0.12	

Power

Groups	Alpha	Power
wt vs Mecp2$^{-/y}$	0.05	<10%

Supplementary Figure 2c
mEPSC amplitude at P15 in wt and Mecp2$^{-/y}$ mice.
Two-tailed t-test.

Groups	n (cells)	N (mice)	Mean ± SEM (normalized to control)	Two-tailed t-test
wt	12	5	18.72 ± 1.73	0.97
Mecp2$^{-/y}$	19	6	18.65 ± 1.14	

Power

Groups	Alpha	Power
wt vs Mecp2$^{-/y}$	0.05	<10%

Supplementary Table 10

Supplementary Figure 3b
mIPSC frequency at P15 in wt and Mecp2$^{-/y}$ mice.
Two-tailed t-test.

Groups	n (cells)	N (mice)	Mean ± SEM (Hz)	Two-tailed t-test
wt	16	6	1.94 ± 0.18	0.058
Mecp2$^{-/y}$	14	7	2.63 ± 0.32	

Power

Groups	Alpha	Power
wt vs Mecp2$^{-/y}$	0.05	0.49

Supplementary Figure 3c
mIPSC amplitude at P15 in wt and Mecp2$^{-/y}$ mice.
Two-tailed t-test.

Groups	n (cells)	N (mice)	Mean ± SEM (normalized to control)	Two-tailed t-test
wt	16	6	29.72 ± 1.85	0.68
Mecp2$^{-/y}$	14	7	30.87 ± 2.05	

Power

Groups	Alpha	Power
wt vs Mecp2$^{-/y}$	0.05	<10%

Supplementary Table 11

Figure 6b
Contribution of bursty cells in wt, Mecp2$^{-/y}$ and bumetanide-pretreated (MP) Mecp2$^{-/y}$ mice.
Fisher's exact test, two-tailed, Bonferroni correction.

n (cells)	n (cells) wt	N (mice) wt	n (cells) Mecp2$^{-/y}$	N (mice) Mecp2$^{-/y}$	Fisher's exact test, two-tailed
Bursty cells	6	3	34	6	0.00016
Non-bursty cells	14		8		
Total	20		42		

n (cells)	n (cells) Mecp2$^{-/y}$	N (mice) Mecp2$^{-/y}$	n (cells) Mecp2$^{-/y}$ bumetanide (MP)	N (mice) Mecp2$^{-/y}$ bumetanide (MP)	Fisher's exact test, two-tailed
Bursty cells	34	6	4	4	$4*10^{-7}$
Non-bursty cells	8		20		
Total	42		24		

n (cells)	n (cells) wt	N (mice) wt	n (cells) Mecp2$^{-/y}$ bumetanide (MP)	N (mice) Mecp2$^{-/y}$ bumetanide (MP)	Fisher's exact test, two-tailed
Bursty cells	6	3	4	4	0.47
Non-bursty cells	14		20		
Total	20		24		

Power

Groups	Alpha	Power
Bursty cells/non-bursty cells ratio: wt vs Mecp2$^{-/y}$	0.05	0.97
Bursty cells/non-bursty cells ratio: Mecp2$^{-/y}$ vs Mecp2$^{-/y}$ bumetanide (MP)	0.05	1.0
Bursty cells/non-bursty cells ratio: wt vs Mecp2$^{-/y}$ bumetanide (MP)	0.05	0.17

Figure 6d
Averaged Pearson's coefficient for dual sEPSC traces at P15 in wt and Mecp2$^{-/y}$ mice
Two-tailed t-test.

Groups	n (pairs of cells)	N (mice)	Mean ± SEM	Two-tailed t-test
wt	4	2	-0.02 ± 0.04	0.03
Mecp2$^{-/y}$	6	5	0.39 ± 0.12	

Power

Groups	Alpha	Power
wt vs Mecp2$^{-/y}$	0.05	0.74

Figure 6g

Bursts parameters at P15 in Mecp2$^{-/y}$ mice and Mecp2$^{-/y}$ mice with acute application of bumetanide.
Paired sample two-tailed t-test.

Groups	n (cells)	N (mice)	Mean ± SEM (normalized)	Paired sample two-tailed t-test
Bursts average area				
Mecp2$^{-/y}$	6	4	1	$2*10^{-4}$
Mecp2$^{-/y}$ bumetanide (acute)	6	4	0.35 ± 0.69	
Bursts amplitude				
Mecp2$^{-/y}$	6	4	1	0.02
Mecp2$^{-/y}$ bumetanide (acute)	6	4	0.53 ± 0.14	
Bursts duration				
Mecp2$^{-/y}$	6	4	1	0.004
Mecp2$^{-/y}$ bumetanide (acute)	6	4	0.43 ± 0.11	
Bursts charge density				
Mecp2$^{-/y}$	6	4	1	0.003
Mecp2$^{-/y}$ bumetanide (acute)	6	4	0.30 ± 0.13	

Power

Parameters	Alpha	Power
Bursts average area: Mecp2$^{-/y}$ vs Mecp2$^{-/y}$ bumetanide (acute)	0.05	1.0
Bursts amplitude: Mecp2$^{-/y}$ vs Mecp2$^{-/y}$ bumetanide (acute)	0.05	0.94
Bursts duration: Mecp2$^{-/y}$ vs Mecp2$^{-/y}$ bumetanide (acute)	0.05	1.0
Bursts charge: Mecp2$^{-/y}$ vs Mecp2$^{-/y}$ bumetanide (acute)	0.05	1.0

Supplementary Table 12

Figure 7d
Early-LTD at P15 in wt, Mecp2$^{-/y}$ and bumetanide-pretreated (MP) Mecp2$^{-/y}$ mice.
One-way ANOVA with Fisher's LSD post-hoc test.

Groups	n (slices)	N (mice)	Mean ± SEM (% of control)
wt	6	3	52.18 ± 1.56
Mecp2$^{-/y}$	6	5	72.62 ± 0.34
Mecp2$^{-/y}$ bumetanide (MP)	7	3	61.72 ± 0.65

Groups	n (slices)	n (slices)	Univariate ANOVA between groups	Fisher's LSD post-hoc test
wt vs Mecp2$^{-/y}$	6	6	$3*10^{-14}$	$1*10^{-14}$
Mecp2$^{-/y}$ bumetanide (MP) vs wt	7	6		$5*10^{-7}$
Mecp2$^{-/y}$ bumetanide (MP) vs Mecp2$^{-/y}$	7	6		$4*10^{-8}$

Power

Groups	Alpha	Power
wt vs Mecp2$^{-/y}$ vs Mecp2$^{-/y}$ bumetanide (MP)	0.05	1.0

Figure 7e
Late-LTD at P15 in wt, Mecp2$^{-/y}$ and bumetanide-pretreated (MP) Mecp2$^{-/y}$ mice.
One-way ANOVA with Fisher's LSD post-hoc test.

Groups	n (slices)	N (mice)	Mean ± SEM (% of control)
wt	6	3	77.83 ± 1.08
Mecp2$^{-/y}$	6	5	75.84 ± 0.34
Mecp2$^{-/y}$ bumetanide (MP)	7	3	87.82 ± 0.34

Groups	n (slices)	n (slices)	Univariate ANOVA between groups	Fisher's LSD post-hoc test
wt vs Mecp2$^{-/y}$	6	6	$5*10^{-13}$	0.0535
Mecp2$^{-/y}$ bumetanide (MP) vs wt	7	6		$4*10^{-11}$
Mecp2$^{-/y}$ bumetanide (MP) vs Mecp2$^{-/y}$	7	6		$4*10^{-13}$

Power

Groups	Alpha	Power
wt vs Mecp2$^{-/y}$ vs Mecp2$^{-/y}$ bumetanide (MP)	0.05	1.0

Supplementary Table 13

Plethysmography experiments in wt, Mecp2$^{-/y}$ and bumetanide-pretreated (MP) Mecp2$^{-/y}$ mice.

Age (weeks)	Age (postnatal days)	wt N(mice)	Mecp2$^{-/y}$ N(mice)	Mecp2$^{-/y}$ bumetanide (MP) N(mice)
3.5 weeks	P24	6	10	8
4 weeks	P28	6	6	8
5 weeks	P35	5	6	8
6 weeks	P42	10	12	7
7 weeks	P49	9	9	5
8 weeks	P56	5	6	7
Total		41	49	43

Power

The statistical power was calculated using a binomial distribution with p=1/3 (corresponding to the number of choices/groups) and number of repetitions equal to the number of animals per group. The p-values calculated from the binomial distribution for the number of animals classified in each group were lower than 10^{-18}.

Supplementary Table 14

Weight changes in wt, Mecp2$^{-/y}$ and bumetanide-pretreated (MP) Mecp2$^{-/y}$ mice.
One-way ANOVA with Bonferroni post-hoc test.

Age (weeks)	wt N(mice)	Mecp2$^{-/y}$ N(mice)	Mecp2$^{-/y}$ bumetanide (MP) N(mice)
3.5 weeks	10	12	11
4 weeks	17	17	12
5 weeks	18	18	12
6 weeks	21	23	10
7 weeks	21	20	8
8 weeks	18	16	10

Mean ± SEM (g)	wt	Mecp2$^{-/y}$	Mecp2$^{-/y}$ bumetanide (MP)
3.5 weeks	10.30 ± 0.56	7.90 ± 0.39	7.44 ± 0.28
4 weeks	14.05 ± 0.68	9.33 ± 0.65	10.63 ± 0.57
5 weeks	18.79 ± 0.39	13.08 ± 0.62	14.46 ± 0.51
6 weeks	21.21 ± 0.32	15.65 ± 0.45	16.48 ± 0.55
7 weeks	22.72 ± 0.25	16.39 ± 0.60	17.09 ± 1.20
8 weeks	23.91 ± 0.36	17.06 ± 0.67	17.14 ± 1.19

3.5 weeks: Groups	Univariate ANOVA between groups	Bonferroni post-hoc test
wt vs Mecp2$^{-/y}$	$8*10^{-5}$	$9*10^{-4}$
Mecp2$^{-/y}$ bumetanide (MP) vs wt		$1*10^{-4}$
Mecp2$^{-/y}$ bumetanide (MP) vs Mecp2$^{-/y}$		1

4 weeks: Groups	Univariate ANOVA between groups	Bonferroni post-hoc test
wt vs Mecp2$^{-/y}$	$1*10^{-5}$	$9*10^{-6}$
Mecp2$^{-/y}$ bumetanide (MP) vs wt		0.003
Mecp2$^{-/y}$ bumetanide (MP) vs Mecp2$^{-/y}$		0.57

5 weeks: Groups	Univariate ANOVA between groups	Bonferroni post-hoc test
wt vs Mecp2$^{-/y}$	$5*10^{-10}$	$5*10^{-10}$
Mecp2$^{-/y}$ bumetanide (MP) vs wt		$4*10^{-6}$
Mecp2$^{-/y}$ bumetanide (MP) vs Mecp2$^{-/y}$		0.25

6 weeks: Groups	Univariate ANOVA between groups	Bonferroni post-hoc test
wt vs Mecp2$^{-/y}$	$2*10^{-13}$	$3*10^{-13}$
Mecp2$^{-/y}$ bumetanide (MP) vs wt		$4*10^{-8}$

$Mecp2^{-/y}$ bumetanide (MP) vs $Mecp2^{-/y}$		0.72

7 weeks: Groups	Univariate ANOVA between groups	Bonferroni post-hoc test
wt vs $Mecp2^{-/y}$	$2*10^{-11}$	$5*10^{-11}$
$Mecp2^{-/y}$ bumetanide (MP) vs wt		$1*10^{-6}$
$Mecp2^{-/y}$ bumetanide (MP) vs $Mecp2^{-/y}$		1

8 weeks: Groups	Univariate ANOVA between groups	Bonferroni post-hoc test
wt vs $Mecp2^{-/y}$	$6*10^{-10}$	$5*10^{-9}$
$Mecp2^{-/y}$ bumetanide (MP) vs wt		$1*10^{-7}$
$Mecp2^{-/y}$ bumetanide (MP) vs $Mecp2^{-/y}$		1

Power

Groups	Alpha	Power
3.5 weeks : wt vs $Mecp2^{-/y}$ vs $Mecp2^{-/y}$ bumetanide (MP)	0.05	0.98
4 weeks : wt vs $Mecp2^{-/y}$ vs $Mecp2^{-/y}$ bumetanide (MP)	0.05	0.98
5 weeks : wt vs $Mecp2^{-/y}$ vs $Mecp2^{-/y}$ bumetanide (MP)	0.05	1.0
6 weeks : wt vs $Mecp2^{-/y}$ vs $Mecp2^{-/y}$ bumetanide (MP)	0.05	1.0
7 weeks : wt vs $Mecp2^{-/y}$ vs $Mecp2^{-/y}$ bumetanide (MP)	0.05	1.0
8 weeks : wt vs $Mecp2^{-/y}$ vs $Mecp2^{-/y}$ bumetanide (MP)	0.05	1.0

Supplementary Table 15

Onset of mortality (Life span in weeks) in wt, Mecp2$^{-/y}$ and bumetanide-pretreated (MP) Mecp2$^{-/y}$ mice.
One-way ANOVA with Bonferroni post-hoc test.

Groups	N (mice)	Mean ± SEM (weeks)
wt	14	32.95 ± 0.71
Mecp2$^{-/y}$	23	10.64 ± 0.54
Mecp2$^{-/y}$ bumetanide (MP)	12	10.63 ± 1.09

<table>
<tr><th>Groups</th><th>Univariate ANOVA between groups</th><th>Bonferroni post-hoc test</th></tr>
<tr><td>wt vs Mecp2$^{-/y}$</td><td rowspan="3">$7*10^{-27}$</td><td>$5*10^{-26}$</td></tr>
<tr><td>Mecp2$^{-/y}$ bumetanide (MP) vs wt</td><td>$2*10^{-23}$</td></tr>
<tr><td>Mecp2$^{-/y}$ bumetanide (MP) vs Mecp2$^{-/y}$</td><td>1</td></tr>
</table>

Power

Groups	Alpha	Power
wt vs Mecp2$^{-/y}$ vs Mecp2$^{-/y}$ bumetanide (MP)	0.05	1.0

Supplementary Figures

Supplementary Figure 1. **KCC2 immunoreactivity is decreased in the CA3 stratum pyramidale of P15 Mecp2$^{-/y}$ mice**. **a** Confocal images of KCC2 overall immunoreactivity in the CA3 stratum pyramidale of wt and Mecp2$^{-/y}$ mice at P15. Scale bar 50 µm. **b** Normalized averaged immunofluorescence of KCC2 in the CA3 stratum pyramidale of wt vs Mecp2$^{-/y}$ mice. The fluorescence intensity was normalized to the average intensity in wt. Dataset analyzed by Mann-Whitney test. Data presented as means ± SEM. ***$p < 0.001$. (see **Supplementary Table 1** for detailed statistics).

Supplementary Figure 2. **mEPSCs in wt and Mecp2$^{-/y}$ mice at P15. a** Representative traces of mEPSCs recorded at -70 mV from individual hippocampal CA3 pyramidal neurons in slices from wt and Mecp2$^{-/y}$ mice at P15. Average values of mEPSC frequencies (**b**) and amplitudes (**c**) in wt and Mecp2$^{-/y}$ mice. Data are presented as mean ± SEM. **b, c** Data were analyzed by Mann-Whitney test and Two-tailed t-test (see Supplementary Table 9 for detailed statistics).

Supplementary Figure 3. **mIPSCs in wt and Mecp2$^{-/y}$ mice at P15**. **a** Representative traces of (a) mIPSCs recorded at -70 mV from individual hippocampal CA3 pyramidal neurons in slices from wt and Mecp2$^{-/y}$ mice at P15. Average values of mIPSC frequencies (**b**) and amplitudes (**c**) in wt and Mecp2$^{-/y}$ mice Data are presented as mean ± SEM. **b, c** Data were analyzed with Two-tailed t-test (see Supplementary Table 10 for detailed statistics).

Supplementary Figure 4. **Breathing abnormality in Mecp2$^{-/y}$ mice is not alleviated by bumetanide. a** Representative plethysmographic recordings showing the irregular rhythm and periods of apnea in Mecp2$^{-/y}$ mice compared to wt. **b** Cumulative probability distributions of inter-breathing cycle-interval (ICI) for each animal at each time point (separated in columns; mice age ranging from P24 to P56) within the three groups (separated in rows) analyzed: wt,

Mecp2$^{-/y}$ and Mecp2$^{-/y}$ mice pretreated with bumetanide (MP). Note that the duration of the recording time analyzed for each animal was the same. The number of animals per condition is described on **Supplementary Table 13**. **c** Similarity between ICI datasets across groups and ages. A color-coded matrix representing the cosine distance between the ICI datasets in each animal pair obtained from the Cliff's Delta matrix (see Methods [102]). The cosine distance matrix was used for hierarchical clustering analysis to identify the three groups of animals in the datasets (i.e. wt, Mecp2$^{-/y}$ and Mecp2$^{-/y}$ + bumetanide (MP)). Broken black lines separate the three groups analyzed, and broken white lines separate different ages. Note that animals within the same group show low distances (corresponding to high similarity between ICI datasets). Also, the distance between Mecp2$^{-/y}$ and Mecp2$^{-/y}$ mice pretreated with bumetanide (MP) animals is low. **d** Results of the hierarchical clustering analysis, which is an unsupervised procedure, aimed at revealing three groups. The cluster one corresponds to the entire wt group, with an additional Mecp2$^{-/y}$ animal. The cluster two corresponds to the vast majority of Mecp2$^{-/y}$ and Mecp2$^{-/y}$ pretreated with bumetanide (MP) animals. The third group is composed by one Mecp2$^{-/y}$ bumetanide (MP) animal. Note that the results of this analysis clearly show that the wt group is statistically different from the Mecp2$^{-/y}$ and Mecp2$^{-/y}$ bumetanide (MP) group. In addition, there is no statistical difference between the Mecp2$^{-/y}$ and the Mecp2$^{-/y}$ bumetanide (MP) group. Statistical significance was verified by random reshuffling of the animal labels prior to dendrogram clustering, through a thousand repetitions (see Methods).

Supplementary Figure 5. **Weight gain deficits and onset of mortality in Mecp2$^{-/y}$ mice is not alleviated by bumetanide. a** Mean values of weight in grams in wt, Mecp2$^{-/y}$ mice, and Mecp2$^{-/y}$ mice with maternal pretreatment (MP) with bumetanide measured at 6 different ages. **b** Mean values of life span in weeks in wt, Mecp2$^{-/y}$ mice, and Mecp2$^{-/y}$ mice with MP bumetanide. Data are presented as mean ± SEM. $^{**}p < 0.01$; $^{***}p < 0.001$ and analyzed by One-way ANOVA with Bonferroni post-hoc test (see **Supplementary Tables 14 and 15** for detailed statistics).

Supplementary Figure 1

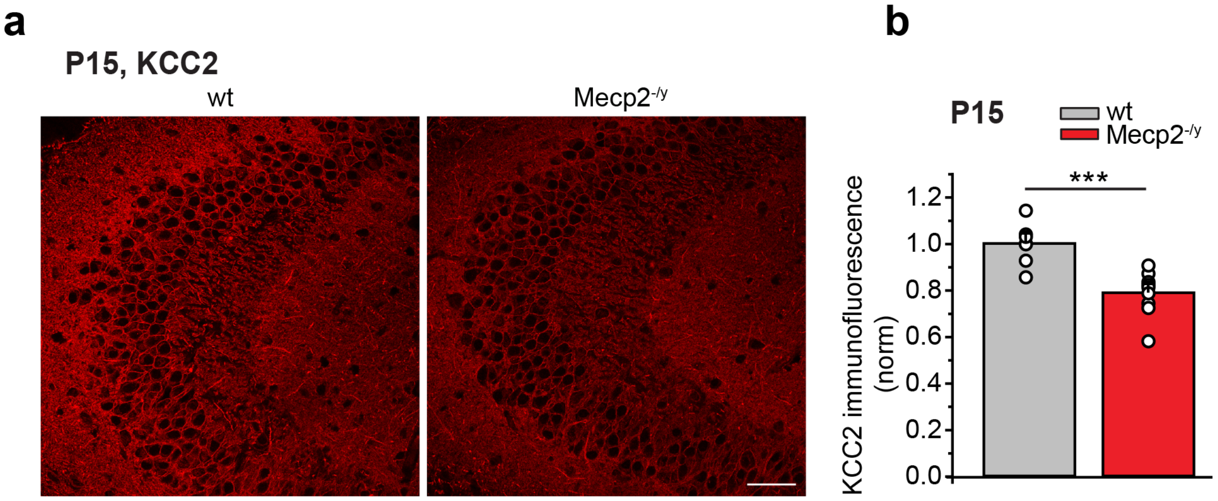

Supplementary Figure 2

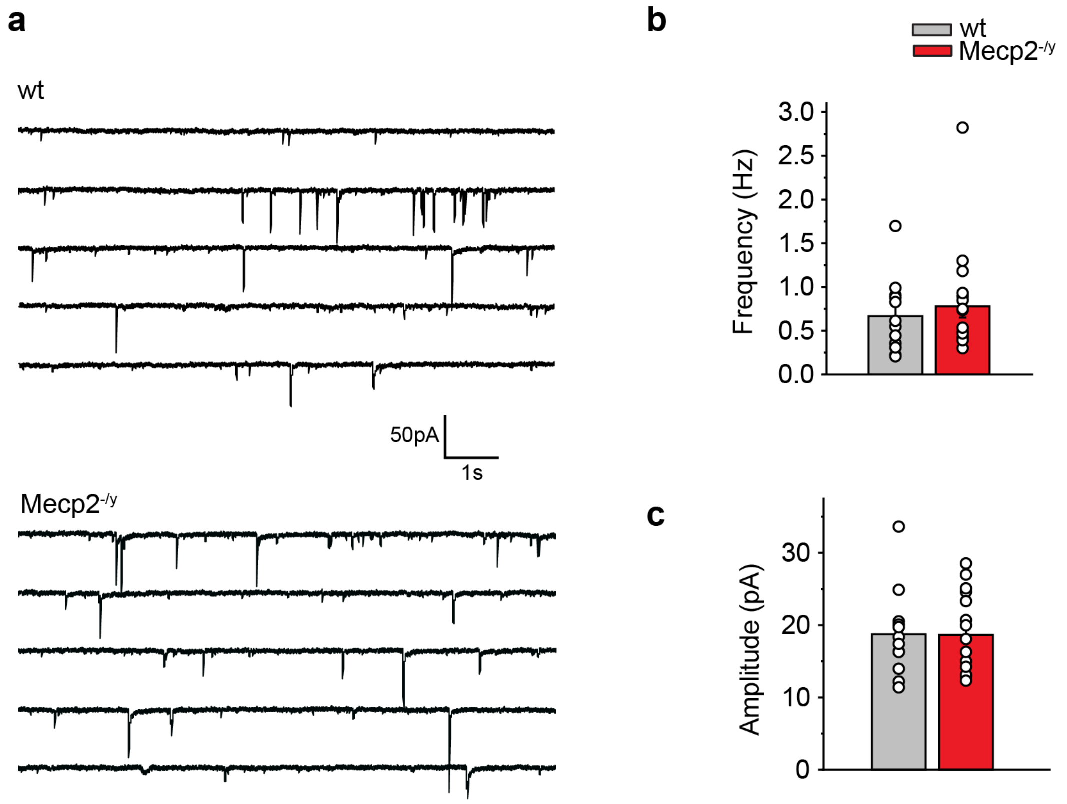

Supplementary Figure 3

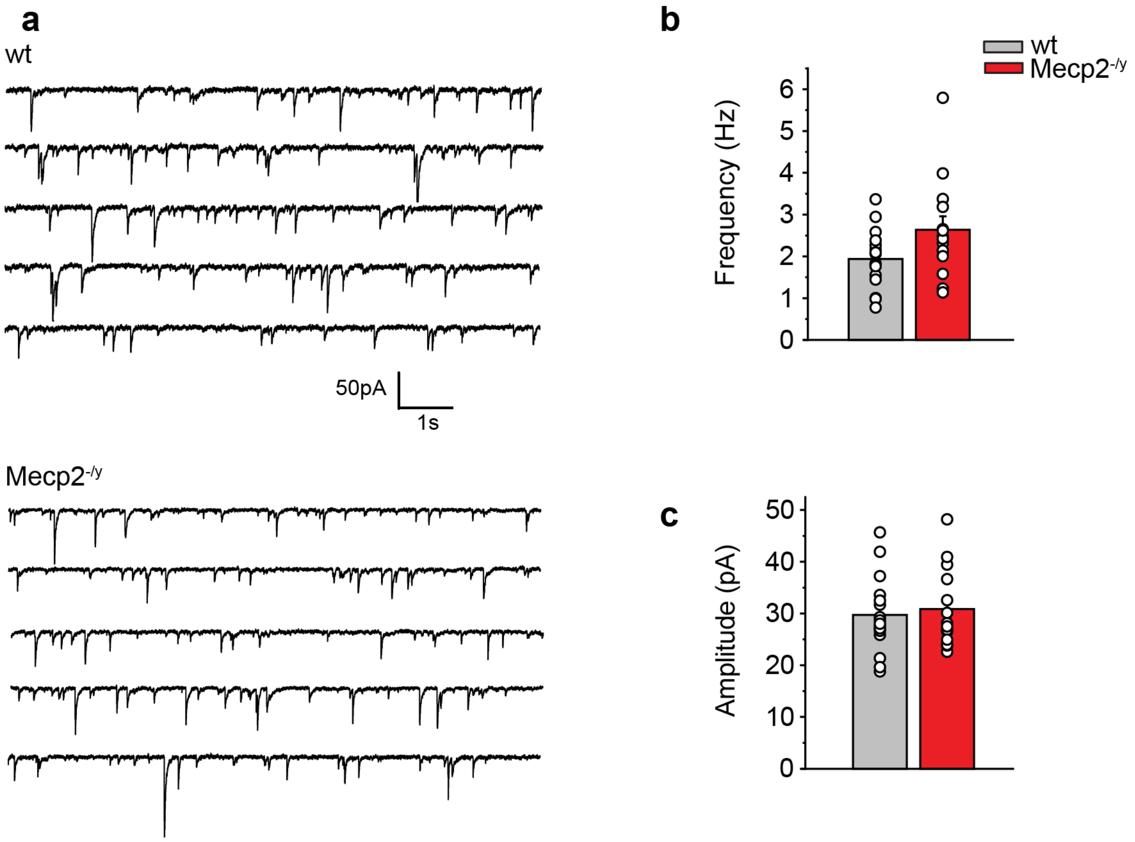

Supplementary Figure 4

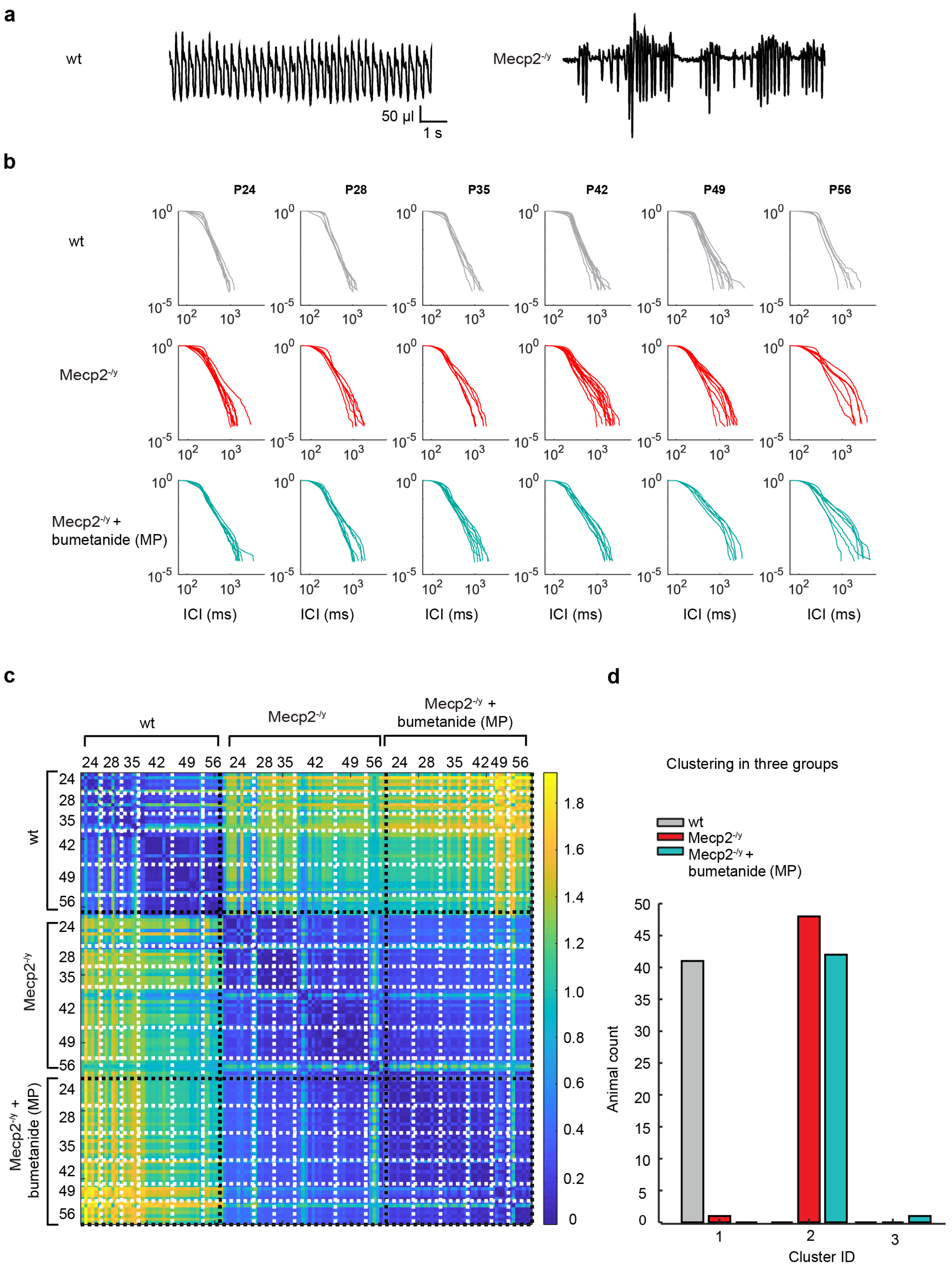

Supplementary Figure 5

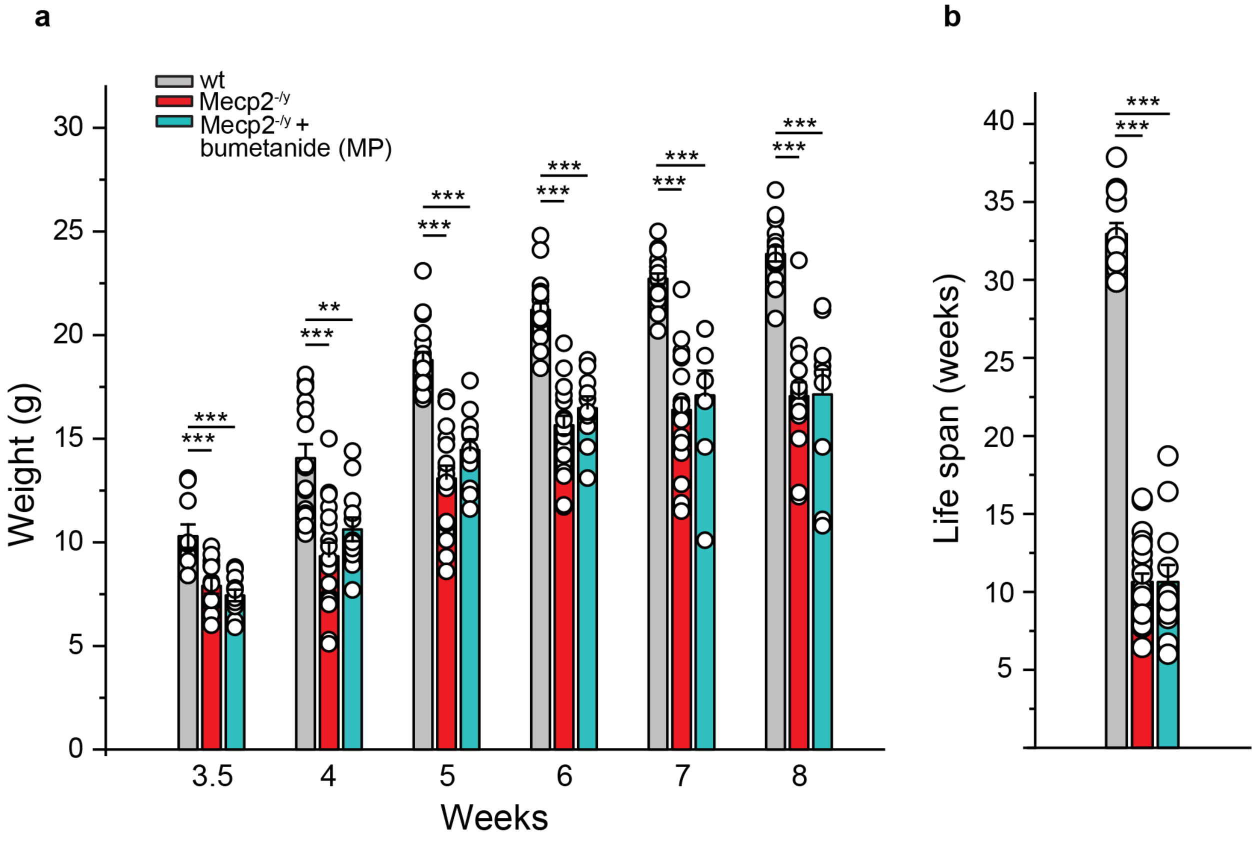

Supplement: Supplementary file 1 — Supplementary materials [file 41598_2019_45635_MOESM1_ESM.pdf]
